# FMNL2 and -3 regulate Golgi architecture and anterograde transport downstream of Cdc42

**DOI:** 10.1038/s41598-017-09952-1

**Published:** 2017-08-29

**Authors:** Frieda Kage, Anika Steffen, Adolf Ellinger, Carmen Ranftler, Christian Gehre, Cord Brakebusch, Margit Pavelka, Theresia Stradal, Klemens Rottner

**Affiliations:** 1 Division of Molecular Cell Biology, Zoological Institute, Technische Universität Braunschweig, Spielmannstrasse 7, 38106 Braunschweig, Germany; 2 Department of Cell Biology, Helmholtz Centre for Infection Research, Inhoffenstrasse 7, 38124 Braunschweig, Germany; 3 Center for Anatomy and Cell Biology, Medical University of Vienna, Schwarzspanierstraße 17, 1090 Vienna, Austria; 4 Biomedical Institute, BRIC, University of Copenhagen, DK-2200 Copenhagen, Denmark

**Keywords:** Actin, Endosomes, Golgi, Lysosomes, Small GTPases

## Abstract

The Rho-family small GTPase Cdc42 localizes at plasma membrane and Golgi complex and aside from protrusion and migration operates in vesicle trafficking, endo- and exocytosis as well as establishment and/or maintenance of cell polarity. The formin family members FMNL2 and -3 are actin assembly factors established to regulate cell edge protrusion during migration and invasion. Here we report these formins to additionally accumulate and function at the Golgi apparatus. As opposed to lamellipodia, Golgi targeting of these proteins required both their N-terminal myristoylation and the interaction with Cdc42. Moreover, Golgi association of FMNL2 or -3 induced a phalloidin-detectable actin meshwork around the Golgi. Importantly, functional interference with FMNL2/3 formins by RNAi or CRISPR/Cas9-mediated gene deletion invariably induced Golgi fragmentation in different cell lines. Furthermore, absence of these proteins led to enlargement of endosomes as well as defective maturation and/or sorting into late endosomes and lysosomes. In line with Cdc42 - recently established to regulate anterograde transport through the Golgi by cargo sorting and carrier formation - FMNL2/3 depletion also affected anterograde trafficking of VSV-G from the Golgi to the plasma membrane. Our data thus link FMNL2/3 formins to actin assembly-dependent functions of Cdc42 in anterograde transport through the Golgi apparatus.

## Introduction

The Golgi apparatus is the central organelle mediating subcellular trafficking of proteins and lipids within the cell. The Golgi stack comprises at least three compartments, the cis-, medial- and trans-Golgi cisternae, each characterized by a specific set of proteins^1–3^. How such an organization into a highly-ordered organelle is accomplished is still poorly understood. Apart from a clear involvement of microtubules (MTs) regarding assembly and morphological maintenance of the Golgi complex as well as secretory trafficking processes, the actin cytoskeleton is also emerging by an increasing amount of data to operate in regulating Golgi morphology and endo- as well as exocytosis. Many actin regulatory proteins, such as Cdc42, WHAMM, WAVE, Arp2/3 complex, cortactin, cofilin, profilin II and several myosins (II, VI, 18, 1b) were found to be associated with the Golgi apparatus and the trans-Golgi network (TGN)^4, 5^. Aside from the Arp2/3 complex, three actin polymerases all belonging to the formin family have so far been linked to Golgi structure, INF2, mDia1 and FMNL1 isoform γ^6–8^. The formin family is a group of actin assembly factors comprising 15 members in mammals^9^. The formin defining features are the formin homology (FH)2 domain, which dimerizes into a donut-shaped ring, binds the fast-growing barbed ends of actin filaments, and mediates formins’ nucleation/elongation activities, and N-terminal to it the adjacent, proline-rich FH1 domain, which recruits profilin-bound actin monomers to fuel actin polymerization^10–13^. The subfamily of formin-like proteins (FMNL) comprises three members, FMNL1 (FRL1), FMNL2 (FRL3) and FMNL3 (FRL2). FMNL proteins belong to the group of Diaphanous-related formins (Drfs), the key feature of which is the presence of N-terminal Diaphanous inhibitory (DID) and C-terminal Diaphanous autoregulatory domains (DAD), the intramolecular interaction of which keeps these formins in inactive states that can be released through binding of small GTPases to formin-specific GTPase-binding domains (GBD) also positioned in the N-terminus^14^.

Cdc42 belongs to the group of Rho-GTPases, all of which function as molecular switches, thereby modulating fundamental cell biological processes such as migration, vesicle trafficking and cytokinesis^15^. Several Rho-GTPases, but in particular Cdc42, have been shown to exert functions at the Golgi apparatus. Interestingly, a pool of endogenous, active Cdc42 is located at the Golgi complex^16–18^, which is thought to regulate and act as reservoir of the plasma membrane pool of this GTPase^19^. In general, Cdc42 has been functionally coupled to vesicle formation at Golgi and TGN, to endo- and exocytosis as well as intracellular trafficking^19, 20^. Specifically however, the Golgi pool has been implicated in polarized membrane trafficking, thus also impacting on establishment and maintenance of cell polarity in migration^19^. Here we report for the first time that all three FMNL members associate with the trans-medial Golgi compartment and various types of vesicles by specific activation and recruitment through the small Rho-GTPase Cdc42. In the present study, we assessed the role of FMNL2 and -3 on Golgi integrity, endosome and lysosome formation as well as anterograde transport of VSV-G protein. Genetic disruption of these formins caused dramatic Golgi dispersal, malformation of vesicular organelles as well as defective anterograde VSV-G transport from the Golgi to the plasma membrane. We identified FMNL formins as new, specific Cdc42 effectors at the Golgi apparatus, thus advancing our understanding of how some of the well-established roles of Cdc42 in trafficking processes can be now directly linked to actin dynamics.

## Methods

### Cell culture and transfections

B16-F1 mouse melanoma cells (ATTC CRL-6323) and NIH3T3 (murine, embryonal fibroblasts; ATTC CRL-1658) were cultured as described^21^. *Cdc42* ^*fl*/−^ as well as *Cdc42* ^−/−^ fibroblastoid cells were cultivated as described^22^. COS-7 cells (ATCC CRL-1651) were cultured in DMEM (4,5 g/l glucose, Thermo Fisher), 2 mM glutamine (Thermo Fisher), 1 mM sodium pyruvate (Thermo Fisher), 1% non-essential amino acids (Thermo Fisher) and 10% FBS (Sigma). B16-F1 cells were transfected using SuperFect transfection reagent (Qiagen), essentially following manufacturer’s instructions. NIH3T3 and *Cdc42* ^*fl/*−^ or *Cdc42* ^−/−^ clones were transfected using JetPrime transfection reagent (Polyplus, VWR). COS-7 cells were transfected with XtremeGene9 (Sigma) according to manufacturer’s guidelines.

### CRISPR/Cas9-mediated genome editing and RNA interference

RNA interference (RNAi) and genome editing of murine B16-F1 and NIH3T3 cells was done as before^21^. The NIH3T3 F2/3 KO clones #9 and #46 described to lack FMNL3 but to still express in-frame deleted variants of FMNL2 at very low dose^21^, were now additionally treated with CRISPR/Cas9 plasmid pSpCas9(BB)-2A-Puro (Addgene plasmid ID:48139) encoding FMNL2 target sequence 5′-GTCATACTGCCGCAGTAGCC-3′, now mediating gene disruption within exon 2. Upon isolation of new individual colonies, their expansion and exploration by Western Blotting, novel clones F2/3 KO #9/10 and F2/3 KO #46/20 completely devoid of FMNL3 and residual FMNL2 protein expression were selected and characterized further in the present study.

### DNA-constructs

EGFP-C1, -C2, -C3 and EGFP-N1, -N2, -N3 were purchased from Clontech (Mountain View, CA, USA), and corresponding mCherry-variants made by replacing EGFP in frame with mCherry. Human FMNL2 cDNA (NM_052905.3; residues 1–1092; isoform b) was as described^21^, and for generation of murine FMNL3 cDNA (NM_¬011711; residues 1–1028), see ref. 21. All truncated variants such as EGFP-FMNL2-FH1-FH2 (residues 518–1026), FMNL2-N-terminus-EGFP (residues 1–517), EGFP-FMNL3-FH1-FH2 (residues 493–968) and FMNL3-N-terminus-EGFP (residues 1–492) were cloned by PCR amplification with respective primers using full length versions as template.

For generation of FMNL1 expression constructs, a cDNA of full length FMNL1 (NM_005892; residues 1–1100; isoform α; UniProt accession number O95466) was kindly provided by Matthias Geyer^21^ (University of Bonn, Germany). For EGFP-tagging, the full length sequence was amplified using forward primer 5′-GAGGAATTCATGGGCAATGCTGCCGG-3′ and reverse primer 5′-GAGGGATCCCTAGTGGTGGTGATGATGG-3′ harboring a stop codon. All generated constructs were sequence verified. Full length expression of all constructs was validated by Western Blotting.

Constitutively active variants of myc-Cdc42-L61 and myc-Rac-L61 were kindly provided by Laura Machesky (Beatson Institute, Glasgow, UK). In order to generate fluorescently-tagged proteins, respective myc-tags were replaced by either EGFP or mCherry. Cdc42 wildtype sequence was subcloned from a pGEX-2T expression plasmid (kindly provided by Alan Hall) into an EGFP-vector, and the latter replaced by mCherry for a red fluorescent version. Human mRFP-1,4-β-galactosyltransferase was initially purchased as pEYFP-Golgi from Clontech, and the EYFP-tag replaced with mRFP. The temperature sensitive VSV-G mutant EGFP-VSV-G (ts045) was as described^23^. Myc-tagged variants of constitutively active Rho-GTPases TC10 (L75), TCL (L79), Wrch (L107), Chp (L89) as well as the fast-cycling mutant of Cdc42 (L28) were kindly provided by Pontus Aspenström (Karolinska Institute, Stockholm, Sweden).

### Site-directed mutagenesis

Quick change site-directed mutagenesis kit (Stratagene) was used to mutate the conserved amino acid isoleucine within the FH2 domain of FMNL2 and FMNL3 into alanine, in order to obtain FMNL mutants lacking actin binding activity. Respective point mutations were introduced into both full length variants and truncated variants (FH1-FH2) of FMNL2 and -3. The PCR reaction was performed according to the Quick change site directed mutagenesis kit manual employing forward primers 5′-AAACAGGGCCAAAAATCTTGCCGCAACTTTAAGGAAAGCTGGAAAG-3′ and 5′-GCCAAGAACTGGCTGCCACCCTTCGCAAGGC-3′ for FMNL2 and FMNL3, respectively, and corresponding complementary sequences as reverse primers.

### Preparation of Cell Extracts, Protein Measurements and Western Blotting

For preparation of detergent-soluble extracts, cells were washed 3x with ice-cold 1x PBS (Gibco), lysed in ice-cold lysis-buffer (140 mM KCl, 50 mM Tris-HCl (pH 7.4), 50 mM NaF, 10 mM Na_4_P_2_O_7_, 2 mM MgCl_2_, 1% Triton-X100, supplemented with one mini complete protease inhibitor pill [Roche]). Finally, cell debris was removed by centrifugation at 20.000 × g for 15 min (4 °C). Soluble protein concentrations were measured using standard Bradford assay. Western Blotting was done according to standard procedures and using primary antibodies as follows: FMNL2/3 (ab57963, Abcam), GFP (101G4B2, self-made), Vinculin (V9131, Sigma), Cdc42 (clone44, BD Biosciences), LAMP1 (1D4B, Abcam), EEA1 (Sc-6415, Santa Cruz), Cathepsin D (kindly provided by Prof. Stefan Höning, Cologne, Germany), GAPDH (6C5, Calbiochem), α-Tubulin (3A2, Synaptic Systems). Secondary antibodies were purchased from Invitrogen.

### Western Blot Intensity Measurements

Detergent-soluble extracts from cells were analyzed with Cathepsin D antibody and compared according to their expression levels of housekeeping genes such as GAPDH. To quantify protein levels, intensity measurements on chemiluminescent images (Intas, Goettingen, Germany) were performed using MetaMorph software. Identical rectangular regions were drawn around protein bands of interest. To correct for background signals contributing to the intensities measured, regions of the same size were measured in background areas close to the band of interest and subtracted from individual, corresponding protein bands. Intensities of measured protein bands were normalized to those of corresponding loading controls. Corresponding data were plotted as bar charts using Excel 2010 (Microsoft).

### Immunoprecipitations and pull down assays

Commercially available GFP-Trap_A (ChromoTek) was used to immunoprecipitate EGFP-tagged proteins. B16-F1 cells were transfected with plasmids encoding EGFP-tagged proteins of interest or EGFP alone as control. Cells were lysed using IP-buffer (140 mM KCl, 50 mM Tris-HCl (pH 7.4), 50 mM NaF, 10 mM Na_4_P_2_O_7_, 2 mM MgCl_2_) supplemented with 1% Triton-X100 and a mini complete protease inhibitor pill (Roche). Lysates were centrifuged at 20.000 × g for 15 min at 4 °C. 10 μl of each cell lysate was mixed with SDS sample buffer referred to as input. 30 μl bead slurry was washed 3x and spinned down (2.500 × g for 2 min at 4 °C). Subsequently, cell lysates were added to the beads and incubated under constant mixing for 1 h at 4 °C. After centrifugation, 10 μl of each sample was incubated with sample buffer and used as control for unbound, EGFP-tagged proteins (supernatant). Finally, beads were washed 3x with IP-buffer prior to addition of 25 μl 8 × SDS sample buffer. All samples were boiled at 95 °C for 5 min prior to loading onto respective SDS-gels. After blotting proteins onto PVDF membranes (Immobilon), respective antibodies (anti-GFP, clone 101G4B2 hybridoma self-made; anti-FMNL2/3, ab57963 Abcam; anti-actin, A3853 Sigma) were used to confirm co-immunoprecipitations of endogenous proteins of interest with overexpressed, EGFP-tagged baits.

For pull downs, recombinant GST- or MBP-tagged Rho-GTPases were expressed and purified as described previously^22^. In case of exploring FMNL1 with respective Rho-GTPases, B16-F1 cells completely devoid of the protein^21^ were transfected with constitutively active EGFP-FMNL1ΔDAD, and subjected to pulldown assays as described^22^. EGFP-FMNL1ΔDAD was detected using a GFP-reactive antibody.

### Immunofluorescence stainings and antibodies

For immunolabelling of the Golgi complex, either a GM130 (610822, BD Biosciences) or a TGN46 (ab16059, Abcam) reactive antibody was used. Early endosomes and lysosomes were visualized using anti-EEA1 (Sc-6415, Santa Cruz) and anti-LAMP1 (1D4B, Abcam) antibodies, respectively. Rab7-positive vesicles were detected with a Rab7- specific antibody (D95F2, Cell Signaling). Prior to fixation and stainings, B16-F1 cells were seeded onto acid-washed glass coverslips that were coated with laminin (25 µg/ml; Sigma) in laminin coating buffer (50 mM Tris, pH 7.4, 150 mM NaCl) for 60 min. In contrast, fibroblasts were replated on fibronectin-coated (25 µg/ml dilution in PBS, Roche, 60 min) coverslips. Cells were allowed to adhere overnight prior to fixation in prewarmed 4% PFA/PBS for 20 min and permeabilization with 0.1% Triton-X100/PBS for 1 min. Upon washing in PBS, cells were blocked with 5% horse serum in 1% BSA/PBS for 30 min, followed by 1 h incubation with respective primary antibodies, washing and incubation with secondary antibodies with or without phalloidin. Alexa Fluor-coupled phalloidin as well as secondary antibodies were purchased from Invitrogen. To stimulate accumulation of endogenous FMNL2 and -3 at the Golgi apparatus, cells were transfected with myc-tagged, constitutively active Cdc42-L61, and stained using a FMNL2/3-reactive antibody (ab57963, Abcam). In order to stain myc-tagged Rho-GTPases, a self-made myc-specific hybridoma supernatant (9E10) was used.

### Electron microscopy

For electron microscopic analyses, different cell lines were fixed in freshly prepared 2% glutaraldehyde ELMI grade in phosphate buffered saline (PBS; both Sigma-Aldrich) for 2 hours at 4 °C, washed and stored in PBS. Subsequently, samples were post-fixed in 1% veronal-acetate-buffered OsO_4_ (Merck) for 1 hour, dehydrated in a graded series of ethanol and embedded in epoxy resin (Serva). Ultrathin sections were stained in 1% uranyl acetate or alternatively, in 0.2% OTE (Oolong Tea Extract, Nisshin EM Co.), and subsequently in 8% alkaline lead citrate (both Merck), followed by examination in a transmission electron microscope at 80 kV (Tecnai 20, FEI Company).

### VSV-G transport assay

Cells transfected with VSV-G-EGFP were seeded onto fibronectin-coated coverslips and allowed to adhere for several hours at 37 °C. In order to retain VSV-G in the ER, cells were transferred to 40 °C (restrictive temperature) overnight. To initiate transport of the VSV-G reporter from the ER to Golgi complex and plasma membrane, cells were relocated to 32 °C (permissive temperature), and fixed after 15 min, 30 min, 1 or 2 h. After washing with warm PBS and fixation with 4% PFA/PBS for 20 min, cells were subjected to phalloidin staining. Time point 0 defined samples fixed immediately after overnight-incubation at 40 °C, thus corresponding to the starting point of the assay.

### Brefeldin A (BFA)-treatment and washout

NIH3T3 cells were seeded onto fibronectin-coated coverslips and allowed to adhere overnight. Coverslips were transferred into a new well containing pre-warmed and pH-equilibrated medium supplemented with 2.5 μg/ml BFA. Cells were incubated at 37 °C for 30 min followed by either immediate fixation with 4% PFA/PBS (“+BFA”) or washout with pre-warmed, regular growth medium for time points as indicated in the figure. Cells without any treatment were used as control (“−BFA”). Cells were fixed and subjected to immunofluorescence stainings as described above (anti-GM130).

### Quantifications

Intensity quantification of VSV-G-EGFP signal in the plasma membrane was performed using MetaMorph software (Molecular Devices Corp., USA). A peripheral, flat region of the cell defined as lamella was chosen to measure membrane-associated signal intensity and an extracellular region was defined as background. Average pixel intensities in background regions were subtracted from average intensities in cellular regions. Net fluorescence intensities of VSV-G-EGFP signals were determined after 60 and 120 min and plotted as bar charts.

In order to objectively estimate the extent of the Golgi dispersal phenotype in different cell lines and various experiments, cells were stained for GM130 to visualize the Golgi apparatus, followed by image acquisition using widefield fluorescence microscopy. Subsequently, images were subjected to automated analysis using the ImageJ plugin “Squassh”^24^, allowing unbiased, computer-aided quantitation of GM130-positive objects discernible per cell. Examples for unbiased object quantification, determined numbers of which are not assumed to be identical but likely correlate with Golgi fragment numbers per cell are displayed in Supplementary Fig. S16.

Quantification of average endosome size was performed using ImageJ plugin “particle analysis” (http://imagej.net/Particle_Analysis) by manually setting appropriate thresholds. Due to strong, nonspecific background fluorescence around the nucleus, only clearly separated individual endosomes were included into quantifications (see threshold images in corresponding figure). Data were plotted as box and whiskers plots using Sigma plot 12.0 (Systat Software).

### Confocal microscopy and Co-localization analysis

Data displayed in Supplementary Figs S7 and S8 were acquired using a spinning disc confocal microscope (Ultra-View VoX, Perkin Elmer). Images were captured with a Nikon eclipse Ti equipped with a Modular Laser system 2.0 (laser lines 488 and 561, Perkin Elmer) using a Plan Apo VC 100x/1.4NA oil immersion objective (Nikon) and an Orca R2 C10600-10B-H camera (Hamamatsu) operated by Volocity 6.2.1 software (Perkin Elmer). Pearson’s correlation coefficient was determined using the co-localization analysis tool (Volocity) by selecting background regions outside of cells for both channels (488 and 561) followed by selection of an ROI (region of interest) comprising FMNL2 or FMNL3 perinuclear signals, respectively. Representative scatter plots were computed using Volocity from the same ROIs.

### 3D – Structured Illumination Microscopy

Data displayed in Supplementary Figs S3, S5 and S18 as well as corresponding Movies 1–3 and 6–11 were acquired on a Nikon SIM-E superresolution microscope equipped with a LU-N3-SIM 488/561/640 laser unit mounted on a Nikon Ti eclipse (Nikon) and a Piezo z drive (Mad city labs). Images were taken with a CFI Apochromat TIRF 100x / 1.49 NA oil immersion objective (Nikon), a Hamamatsu Orca flash 4.0 LT camera and an N-SIM motorized quad band filter combined with N-SIM 488 and 561 bandpass emission filters using laser lines 488 and 561 driven by NIS-Elements software (Nikon). Reconstructions were performed with the stack reconstruction tool (Nikon, NIS-Elements).

### Live cell imaging

Light microscopy was performed on an inverted Axiovert S100TV (Carl Zeiss) epifluorescence microscope equipped with electronic shutters (Uniblitz Electronic 35 mm including driver Model VMMD-1, BFI Optilas), a filter wheel (LUDL Electronic products LTD), filter cubes (Chroma Technology Corp. Rockingham), epifluorescence illumination (light source HXP 120, Zeiss), and a tungsten lamp (Osram, HLX64625, FCR 12 V, 100 W) for phase contrast optics. Cells were observed in an open chamber (Warner Instruments) with a heater controller (Model TC-324 B, SN 1176) at 37 °C. Ham’s F12 medium containing all growth supplements was used for imaging. Live cell imaging was performed using 63x/1.4-NA or 100x/1.4-NA plan apochromatic objectives. Images were acquired with a back-illuminated, cooled, charge-coupled-device (CCD) camera (CoolSnap HQ2, Photometrics) driven by VisiView software (Visitron Systems).

### Data Processing and Statistical Analyses

Brightness and contrast levels were adjusted using MetaMorph software. Figures were further processed and assembled with Photoshop CS4. Data analyses were carried out in ImageJ and MetaMorph, Excel 2010 and Sigma plot 12.0. Datasets were compared using either the non-parametric Mann-Whitney rank sum test or unpaired t-test (Sigma plot 12.0). A probability of error of 5% (p ≤ 0.05; * in figure panels) was considered to indicate statistical significance. ** and *** indicated p-values ≤ 0.01 and ≤ 0.001, respectively.

### Data availability

The datasets generated during and/or analysed during the current study are available from the corresponding author on reasonable request.

## Results and Discussion

### All FMNL members localize to the *trans*-medial Golgi compartment

**Figure 1:**
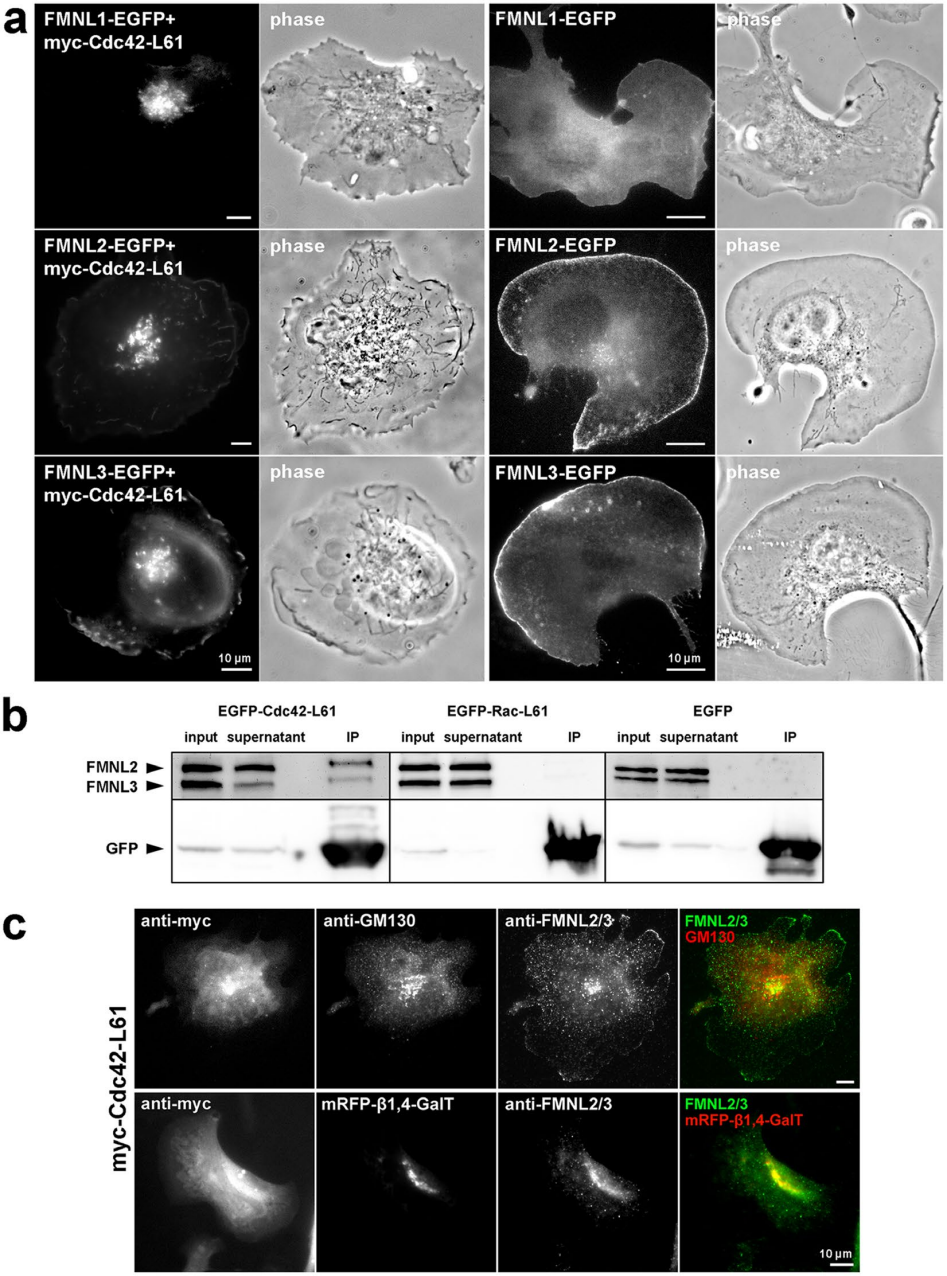
FMNL formins localize to the Golgi apparatus upon interaction with active Cdc42. (**a**) Epifluorescence and corresponding phase contrast images of murine B16-F1 melanoma cells transfected with C-terminally tagged, full length FMNL1, -2 and -3 with or without constitutively active Cdc42, as indicated. Pancake-shape cell morphologies are indicative of Cdc42 expression. Note the perinuclear accumulation of FMNL members in membranous organelles, which clearly coinicides with Cdc42 expression. (**b**) Co-immunoprecipitation experiments of overexpressed EGFP-tagged Cdc42-L61, -Rac1-L61 or EGFP alone employing a commercially available GFP-trap; GFP-antibody documents amounts of respective, ectopically expressed protein in each cell fraction, as indicated. Anti-FMNL2/3 antibody detected co-precipitation with EGFP-tagged Cdc42-L61, but not with Rac-L61 or EGFP alone. (**c**) Endogenous FMNL2 and -3 colocalize with trans-medial Golgi. Endogenous FMNL formins were targeted to the Golgi by myc-tagged Cdc42-L61, the expression of which as verified by anti-myc staining (left panel). Anti-GM130 staining and mRFP-β1,4-galactosyltransferase were used as markers for the cis-Golgi compartment (top) and trans-medial Golgi cisternae (bottom), respectively.

It was previously reported that FMNL1 isoform γ enriches in perinuclear puncta co-localizing with the *cis*-Golgi marker GM130 and the *trans*-Golgi marker Golgin 97. In addition, the authors observed FMNL1 dispersal following brefeldin A (BFA) treatment, confirming FMNL1 to constitute a Golgi-associated protein^6^. We thus tested whether accumulation at intracellular, membranous organelles is specific to FMNL1γ or a feature shared by various FMNL isoforms and family members. Indeed, we and others have shown recently that both FMNL2 and -3 are regulated by the small Rho-GTPase Cdc42, also established to localize and function at the Golgi apparatus^19^. Consequently, we expressed C-terminally tagged, full-length FMNL1 (isoform α), FMNL2 and FMNL3 either alone or together with constitutively active Cdc42-L61, and explored their respective subcellular localizations in each experimental condition (Fig. 1a). Interestingly, all three FMNL formin subfamily members were capable of prominently associating with perinuclear structures strongly reminiscent of the Golgi apparatus upon co-transfection of Cdc42-L61, in addition to their well-known targeting to lamellipodia tips in case of FMNL2 and -3 (refs 21 and 22), which was less intense in comparison in cells harboring prominent perinuclear accumulation (Fig. 1a). However, over-enhancing of such images clearly revealed the simultaneous presence of EGFP-tagged FMNL2 and -3 at the lamellipodium tip, in spite of being non-detectable upon contrast adjustment to Golgi-resident protein (Supplementary Fig. S1). Without co-expression of Cdc42-L61, targeting to lamellipodia tips of FMNL2 and FMNL3 was more evident than their perinuclear accumulation in the majority of cells^21, 22^, while the less closely related FMNL1 displayed a general association to the plasma membrane without specific accumulation in the lamellipodium (Fig. 1a), as expected^25^. Importantly, there is a striking overlap between each of the FMNL members tagged to EGFP and mCherry-Cdc42 in perinuclear regions as well as on vesicular structures (Supplementary Fig. S2). This was also confirmed by superresolution microscopy using a 3D-Structured Illumination Microscope (3D-SIM) (Supplementary Fig. S3, Supplementary Movie 1).

Notably, however, since Cdc42-L61 is considered to be GTPase-defective, hence locked in its GTP-bound state, we also explored effects on FMNL family member localization exerted by additional Cdc42 variants, including constitutively active Cdc42-L28, which can undergo spontaneous GTP for GDP exchange while maintaining full GTPase activity^26^, as well as wildtype Cdc42. Importantly, co-expression of FMNL2- or FMNL3-EGFP with all these Cdc42 variants was able to induce their accumulation in perinuclear, Golgi-like regions, frequently coinciding with Cdc42 variant accumulation (Supplementary Fig. S4). Thus, we conclude that targeting of FMNL subfamily formins to the Golgi by Cdc42 is not restricted to the constitutively active, GTPase-locked variant of this GTPase.

Finally, using superresolution microscopy (3D-SIM) of transfected COS7-cells, we could also detect the accumulation of FMNL2- or FMNL3-EGFP near the *cis*-Golgi marker GM130 (see also below), confirming that depending on conditions and cell type, endogenous Cdc42 can drive the accumulation of expressed EGFP-tagged variants in this perinuclear location (Supplementary Fig. S5, Supplementary Movies 2 and 3).

In addition to this localization at the Golgi apparatus, both FMNL2 and-3 can be found at various types of vesicles of different sizes (Supplementary Movies 4 and 5). Furthermore, EGFP-tagged Cdc42-L61 but not EGFP-Rac1-L61 or EGFP alone was capable of co-precipitating endogenous FMNL2 and FMNL3 (Fig. 1b), both expressed and detectable with the same antibody in B16-F1 cells (Fig. 1b and ref. 22). This was consistent with lack of interaction of FMNL2 with constitutively active Rac1 in ITC assays *in vitro* ^22^, and the failure to release autoinhibitory interactions of EGFP-FMNL2 and –FMNL3 *in vivo* ^21, 22^. As shown previously, FMNL1 is not endogenously expressed in B16-F1 melanoma cells^21^. However, and consistent with biochemical and structural data^27^, an active FMNL1 variant ectopically expressed in cells could also only be precipitated by GST-tagged, constitutively active Cdc42, but not by dominant negative Cdc42 (N17) or corresponding variants of Rac1, nor by constitutively active RhoA or Rif (Supplementary Fig. S6), although identical preparations of the two latter had previously been confirmed to display binding to their common interaction partner mDia1^22^. Taken all this together, we conclude that all three FMNL subfamily members can be equally stimulated to target to the Golgi by Cdc42.

The Golgi apparatus possesses a complex organization characterized by distinct compartments depending on unique protein compositions. According to this specialized protein repertoire, each compartment exerts distinct, specific functions. To explore whether FMNL formins associate with a specific subcompartment of the Golgi, we co-expressed FMNL2-EGFP with myc-tagged Cdc42-L61 in combination with co-expression or endogenous staining of different Golgi markers allowing discrimination between *cis*- versus *trans*-medial Golgi regions or the *trans*-Golgi network (TGN), followed by analysis of the samples by spinning disc confocal fluorescence microscopy (Supplementary Fig. S7a). The data revealed FMNL2-EGFP to accumulate in perinuclear regions close to but not overlapping with the *cis*-Golgi marker GM130, consistent with superresolution data described above (Supplementary Fig. S5). A similar conclusion was drawn from overlaying FMNL2-EGFP images with the trans-Golgi compartment marker TGN46. In contrast, significant overlap could be observed with 1,4-β-galactosyltransferase, defining the *trans*-medial Golgi compartment (Supplementary Fig. S7). Scatter plots of green *versus* red fluorescence intensities (Supplementary Fig. S7b) and statistical analyses of Pearson’s correlation coefficients for the different stainings confirmed the view that the best overlap in these images could be obtained for FMNL2-EGFP and the trans-medial Golgi. Similar results were obtained for FMNL3-EGFP (Supplementary Fig. S8), revealing that the Pearson’s correlation coefficient for the FMNL3 and 1,4-β-galactosyltransferase comparison was even higher than that seen for FMNL2 (compare Supplementary Figs S7c and S8c). The same conclusion was drawn from widefield imaging of respective Golgi compartment markers and EGFP-tagged FMNL2, FMNL3 or FMNL1α (data not shown). Together, all these data clearly establish a principal capability of FMNL formins to accumulate at the Golgi, in tight association with its preferred small GTPase Cdc42^21, 22, 27^. Notably, Cdc42-L61 triggered prominent Golgi positioning of EGFP-tagged FMNL formins only in a fraction (roughly one third) of transfected cells. However, Golgi accumulation upon Cdc42 expression and its clear co-localization with the expressed GTPase was also seen for endogenous FMNL2 and-3 (Fig. 1c), confirming the data obtained with fluorescently tagged FMNL variants (see above). And again, endogenous FMNL2/3 co-localized with galactosyltransferase rather than with GM130 (Fig. 1c).

### Cdc42-induced FMNL2/3 accumulation stimulates formin-specific actin filament assembly

In previous work, we established that FMNL formins, restricted in expression to FMNL2- and 3 in B16-F1 melanoma cells, promote actin assembly in and force generation by lamellipodia downstream of Cdc42^21^. Importantly, phenotypes were highly comparable upon concomitant suppression of FMNL2/3 expression in these cells by RNA interference *versus* functional elimination of both genes using CRISPR/Cas-mediated genome editing. However, FMNL2/3 null cell lines derived upon CRISPR/Cas-mediated gene disruption not only proved useful for loss of function studies, but also for exploring mediators of subcellular distribution and regulation of these formins (see also below). This is because in cells expressing endogenous FMNL variants, which as all Diaphanous–related formins display autoregulatory interactions and operate as dimers^14, 28^, functional and localization studies of specific, ectopically expressed formin variants are complicated by potential dimerization with endogenous proteins, as FMNL2/3 are even described to be capable of forming heterodimers^29^.

**Figure 2:**
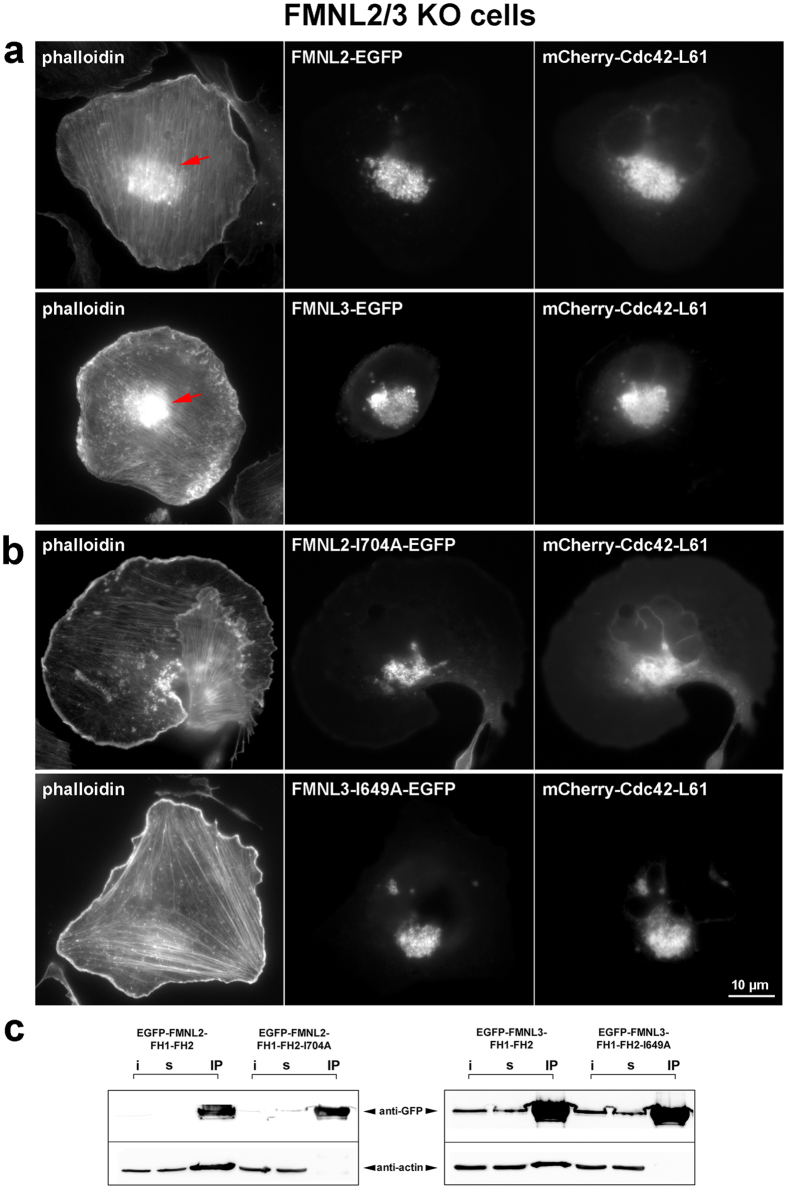
Active FMNL2 and -3 polymerize an F-actin meshwork around the Golgi complex. (**a**,**b**) B16-F1 cells devoid of endogenous FMNL2/3 expression were transfected with either C-terminally tagged full length FMNL variants (**a**) or identical constructs except for harboring a point mutation known to disrupt FH2 dependent F-actin binding (**b**). Note prominent phalloidin-stainable F-actin meshwork around the Golgi apparatus in the region where the formin is located (**a**, red arrows). Respective F-actin binding mutants were still recruited to the Golgi, but failed to induce such an actin network (**b**). (**c**) Co-immunoprecipitation of endogenous actin with FH1-FH2 formin fragments with or without I→A mutations, as indicated, confirming that they abrogated F-actin binding. i = input, s = supernatant, IP = immunoprecipitation fraction.

Actin filaments are thought to contribute to the maintenance of the flattened shape of Golgi cisternae^4, 30^, and can facilitate membrane deformations driving processes as various as vesicle formation, scission and fusion. However, specific staining of actin filaments at the Golgi using phalloidin appeared challenging in the past^31^. To test whether recruitment of fluorescently-labeled FMNL2 or FMNL3 to the Golgi upon co-expression of Cdc42 coincided with actin accumulation, cells co-overexpressing mCherry-tagged, constitutively active Cdc42 (L61) and EGFP-tagged FMNL formins were fixed and counterstained with Alexa350-labeled phalloidin (Fig. 2a). Both overexpressed proteins strongly co-accumulated at the cell centre previously defined to constitute the trans-medial Golgi compartment (see above), which in addition displayed prominent phalloidin staining (arrows in Fig. 2a). To prove these actin accumulations to depend on the respective, overexpressed formin, we mutated an isoleucine, which is conserved in all formins and considered essential for actin binding of their FH2-domains, to alanine^32–35^. Importantly, respective FMNL2 and FMNL3 mutants (FMNL2-I1704A and FMNL3-1649A, respectively) still co-localized with Cdc42, but failed to induce the prominent actin accumulations frequently seen with wildtype FMNL2 or FMNL3 (Fig. 2b). Co-immunoprecipitation experiments employing parts of the C-termini of these formins (FH1-FH2) with or without the corresponding I to A mutation confirmed actin binding of respective wildtype sequences, but complete abolishment of interactions with actin in case of the mutants (Fig. 2c). Similar results were obtained using respective full-length formins (data not shown). Thus, actin accumulation appeared to require direct interactions of Golgi-targeted FMNL formins with actin. This conclusion is consistent with quantifications of actin accumulations in those cells displaying prominent accumulation of EGFP-tagged FMNL3 (Supplementary Fig. S9). Golgi-associated actin meshworks were formed in ~70% of the cells also displaying FMNL3-EGFP accumulation at these sites, whereas no incidence of actin accumulation could be detected in cells in which FMNL3-EGFP localization was restricted to the cell periphery (Supplementary Fig. S9). We thus conclude phalloidin-stained actin filaments in these conditions to derive from direct, FMNL2 or -3-induced polymerization, fully compatible with biochemical activities recently established *in vitro* ^21^.

### Golgi and lamellipodial accumulations of FMNL formins are mediated by distinct mechanisms

We have shown recently that FMNL2 and FMNL3 but not FMNL1 (ref. 25) localize to lamellipodia and filopodia tips upon activation by Cdc42, thereby regulating cell migration^21, 22^. Importantly, Cdc42 triggered activation of these formins through releasing them from autoinhibition, but not through physically contributing to recruitment of these formins to the lamellipodium tip^22^. This was confirmed by the lamellipodial targeting of C-terminal FMNL2 or -3 variants lacking the GTPase-binding domain entirely (ref. 25 and unpublished data), and by the fact that the N-terminus of FMNL2 still harboring the GTPase-binding domain but lacking the C-terminus was not able to target to lamellipodia. Nevertheless, such an FMNL variant retained its capability to interact with the plasma membrane, directly through N-terminal myristoylation as well as a basic patch and indirectly through Cdc42^27^. Thus, interactions with Cdc42 drive activation, i.e. release from autoinhibitory, intramolecular interactions of FMNL formins, operating as prerequisite of lamellipodial targeting in case of FMNL2 and FMNL3, but do not mediate lamellipodial targeting directly (see model in ref. 22).

To explore whether Cdc42 can drive the formin to the Golgi, independent of lamellipodial targeting and/or actin binding mediated through the C-terminus of FMNL2 and FMNL3, we co-expressed mCherry-Cdc42-L61 with the EGFP-tagged N-terminal halves of FMNL2 or FMNL3, comprising the GTPase-binding domains, which readily co-localized with Cdc42 in the plasma membrane and/or in vesicles and an intracellular structure reminiscent of the Golgi (Supplementary Fig. S10). The latter association was never seen in cells expressing the N-termini of FMNL2 or -3 alone (data not shown). Note that due to the use of B16-F1 cells genetically disrupted for FMNL2 and -3 and lacking endogenous FMNL1^21^, potential recruitment of ectopically expressed, tagged proteins through dimerization with endogenous proteins could again be excluded (Supplementary Fig. S10).

To test whether Golgi targeting by Rho-GTPase co-expression is a specific feature of Cdc42, or might also be mediated by additional, related GTPases, we co-expressed full length FMNL2 (Supplementary Fig. S11) or FMNL3 (Supplementary Fig. S12) with constitutively active variants of Cdc42 (as control) or Rac1 or closely related Cdc42-like Rho-GTPases of which at least some are known to localize to the Golgi (Supplementary Figs S11, S12; red arrows). However, neither active Rac1 nor any of the Cdc42-like Rho-GTPases (TC10, TCL, Wrch and Chp) were able to induce Golgi association of FMNL2 (Supplementary Fig. S11) or FMNL3 (Supplementary Fig. S12). Identical results were obtained with FMNL1-EGFP (data not shown). Thus, and at variance to lamellipodial accumulation of FMNL2 and -3, these data indicate that interactions with Cdc42 directly and specifically contribute to accumulation at intracellular vesicular structures and Golgi.

Moreover, we also explored if membrane association through myristoylation might contribute to Golgi targeting of FMNL2 and -3. N-terminal myristoylation at glycines 2 of FMNL2 and -3 can be simply blocked by N-terminal tagging, e.g. with EGFP^22^. Interestingly, when co-expressing in FMNL2/3 double-KO B16-F1 cells Cdc42-L61 with FMNL2 or -3 fused to EGFP either at their N- or C-termini, we found that as opposed to C-terminal tagging allowing N-terminal myristoylation, N-terminally-tagged constructs were never seen to target to the Golgi (Supplementary Fig. S13). Again, the latter constructs could still be readily found at the tips of lamellipodia, due to activation by constitutively active Cdc42 (L61) (Supplementary Fig. S13, red arrowheads), as shown previously^21, 22^, thus the failure to target to the Golgi cannot be explained by incomplete activation. The same result was obtained with a point mutation in FMNL2 also prohibiting myristoylation (G2A)^22^, precluding that lack of Golgi association with EGFP-FMNL2 was due to adverse effects of N-terminal EGFP-tagging other than block of myristoylation (Supplementary Fig. S13, bottom panel). We conclude therefore that as opposed to lamellipodial targeting, N-terminal myristoylation also directly contributes to Golgi positioning of FMNL2 and FMNL3.

### RNA interference as well as genetic deletion of FMNL2 and -3 cause Golgi complex dispersal

In order to determine a possible role of FMNL2 and -3 in Golgi morphology and integrity, expression of both formin proteins was reduced by RNA interference (RNAi) in B16-F1 cells or eliminated entirely in both B16 cells and NIH3T3 fibroblasts using the CRISPR/Cas9 system. As described previously, FMNL1 is not detectably expressed in either cell type^21^. Consistent with observations by Colon-Franco and colleagues^6^, individual knockdown of FMNL2 or FMNL3 by RNAi had little effects on Golgi integrity (Supplementary Fig. S14). However, combined knockdown of both was found to cause significant alteration of Golgi morphology (Supplementary Fig. S14), indicating that FMNL2 and -3 functions at the Golgi might be at least partially redundant. More specifically, combined knockdown of FMNL2 and -3 caused increased and decreased frequencies of cells displaying a dispersed *versus* compact Golgi, respectively. In contrast, cell numbers with an intermediate, spread Golgi morphology remained comparably constant. Consistently, B16-F1 cells eliminated in expression for FMNL2 and -3 by CRISPR/Cas9-mediated gene disruption showed a phenotype highly similar in extent to RNAi data (Supplementary Fig. S15). Aside from qualitative assessments of Golgi architectures, we sought to define more quantitative parameters of Golgi dispersal in these widefield fluorescence microscopy images. As one parameter, we counted separable GM130-positive objects found in each cell in a computer-aided fashion (see Methods and Supplementary Fig. S16), likely correlating with but not necessarily equal to numbers of *cis*-Golgi fragments per cell discernible by fluorescence microscopy upon GM130 staining. Importantly, average cis-Golgi objects were more than doubled in both cell lines lacking FMNL2 and -3 (Supplementary Fig. S15), clearly confirming aforementioned phenotypes using subjective assessment of Golgi morphologies.

Altered Golgi architectures upon loss of FMNL2/3 in B16-F1 mouse melanoma cells were also validated by electron microscopy (Supplementary Fig. S17). Notably, Golgi apparatus morphologies were quite variable in both control and FMNL2/3 KO cells. However, the predominant phenotype of control cells constituted an extended Golgi region close to the nucleus, with ribbons arranged as parallel, well ordered stacks of cisternae often surrounded by moderate amounts of vesicles. By contrast, in the majority of the KO clone cells, Golgi architectures appeared less organized. More specifically, distinct Golgi regions were missing and connected Golgi ribbons were rarely observed. Instead, mini-stacks of short and loosely organized cisternae and contorted membranes surrounded by vesicles were dispersed in the cytoplasm. More regularly organized stacks of cisternae and disorganized mini-stacks could be found located side by side (Supplementary Fig. S17).

**Figure 3:**
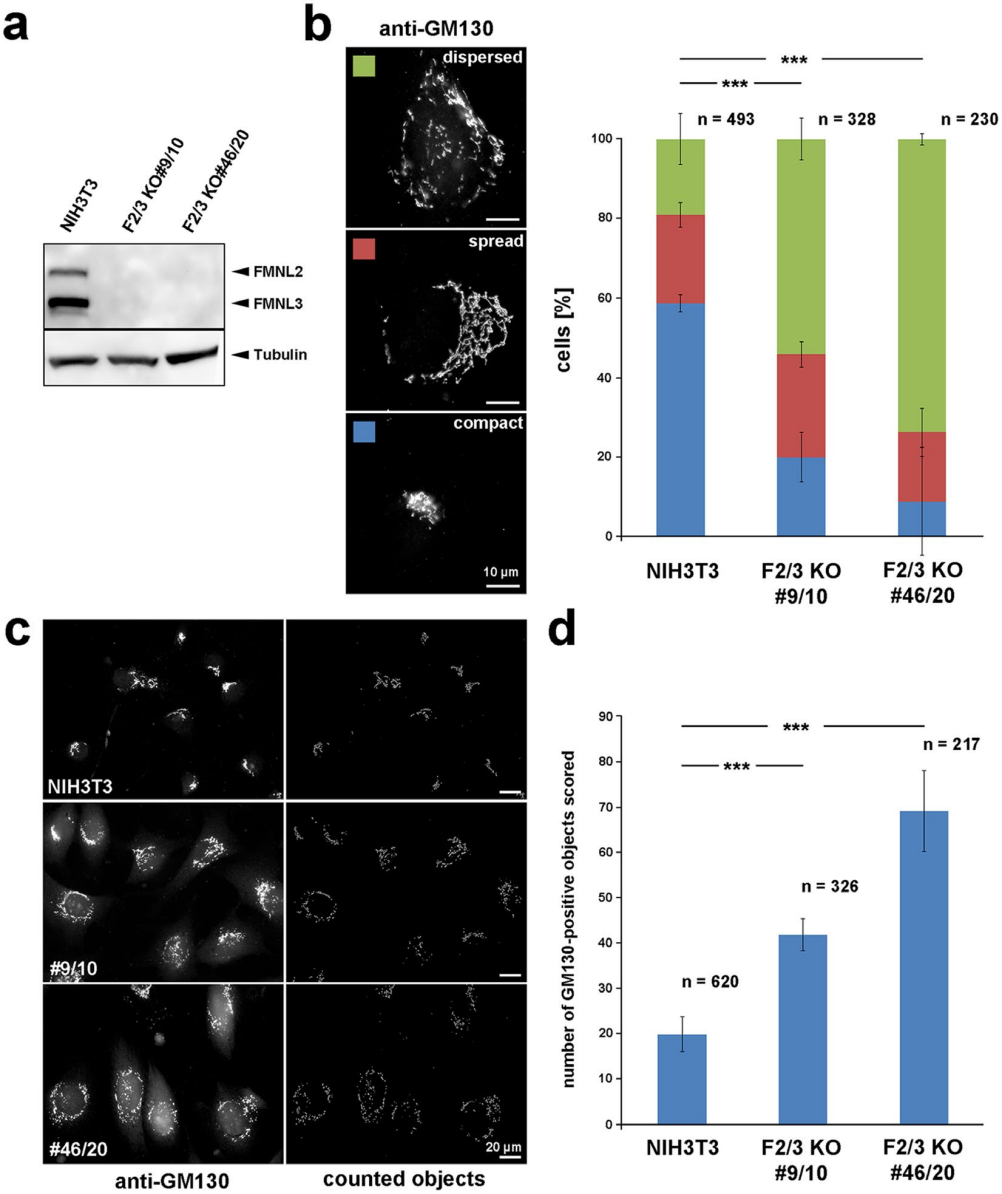
Genetic disruption of FMNL2 and -3 expression alters Golgi morphology. (**a**) CRISPR-Cas9-mediated gene disruption of FMNL2 and -3 expression in two clones (NIH3T3 fibroblasts) as validated by Western Blotting using a FMNL2/3 reactive antibody. Tubulin was used as loading control. (**b**) Analysis of Golgi morphologies in control fibroblasts (NIH3T3) and two FMNL2/3 KO clones. The Golgi-morphologies of cells were manually categorized upon anti-GM130 staining as compact, spread or dispersed, as exemplified on the left. Right panel: Percentages of cells displaying corresponding Golgi phenotypes. Datasets are represented as stacked columns with standard errors of means (three independent experiments). Differences in frequencies of the dispersed phenotype were confirmed to be statistically significant (t-test). (**c**) Representative examples of NIH3T3 control cells and two FMNL2/3 KO clones stained for GM130 (left) and outlines of objects identified and counted using Image J (right panel). (**d**) Bar chart showing average number of GM130-positive objects scored per cell, using the ImageJ Squassh tool. Error bars correspond to standard errors of means (sem) and n is number of analyzed cells from three independent experiments. Datasets were statistically compared using the non-parametric Mann-Whitney rank sum test.

We also studied Golgi organization upon FMNL2/3 gene removal in NIH 3T3 fibroblasts. Previously characterized CRISPR/Cas-treated cell lines were deficient for FMNL3 but still harbored a very low dose of FMNL2^21^. To eliminate FMNL2 expression completely, FMNL2 was once again disrupted using an additional, distinct targeting sequence (see Methods), resulting in complete elimination of gene expression (Fig. 3a). Interestingly, resulting lines displayed a dramatically increased percentage of cells harboring a dispersed Golgi (Fig. 3b), coinciding with more than a doubled or tripled number of software-counted *cis*-Golgi objects, depending on the KO cell line used (Fig. 3c,d). To explore whether the increase of GM130-stained objects in widefield fluorescence microscopy indeed reflected an increase in Golgi fragments in these cells, we also performed superresolution microscopy of the three distinct GM130-stained NIH 3T3 cell lines (Supplementary Fig. S18), clearly establishing the average number of individual, *cis*-Golgi fragments in these representative examples to be increased in the absence of FMNL family proteins as compared to NIH 3T3 controls (see also Supplementary Movies 6–11, showing two representative 3D- reconstructions of *cis*-Golgi compartments for each cell line).

**Figure 4:**
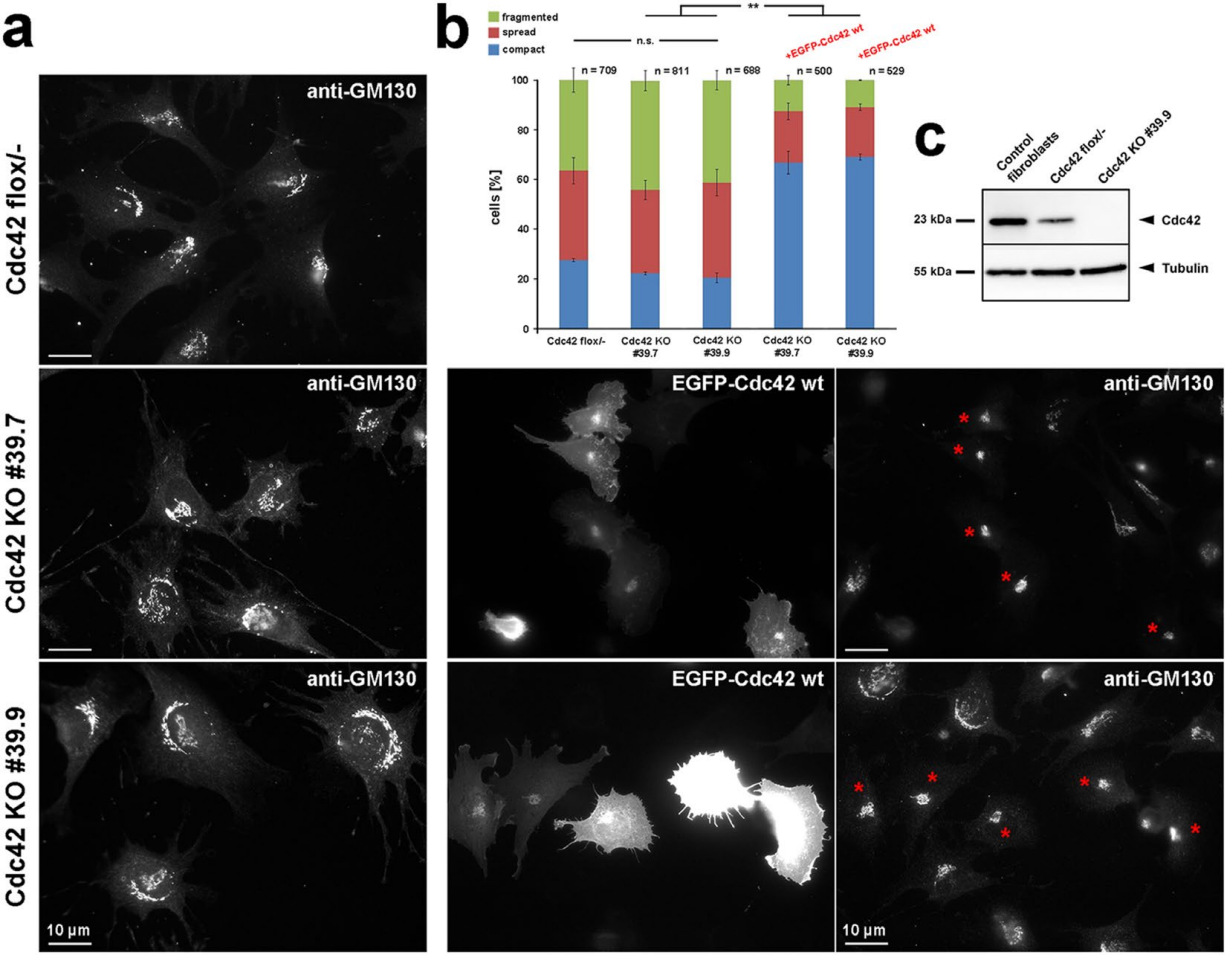
Cdc42 expression levels affect Golgi morphology. (**a**) Representative examples of Cdc42 flox/- fibroblastoid cells and two Cdc42 KO clones stained for the cis-Golgi compartment using GM130-reactive antibody (left column). Moreover, both KO clones were transfected with EGFP-tagged wildtype Cdc42 (middle panel), with representative transfected cells displaying a compact Golgi as judged by GM130 staining highlighted by red asterisks in panels on the right. (**b**) Bar graph displaying percentages of cells harboring Golgi morphologies as classified in Fig. 3a (compact, spread, fragmented). Columns 4 and 5 show classification of observed Golgi morphologies of both Cdc42 KO clones transfected with EGFP-tagged, wildtype Cdc42; n gives number of cells analyzed, error bars are ± sem from three independent experiments. Differences in cell numbers exhibiting a dispersed Golgi morphology were not statistically significant when comparing flox/- control cells with each of the KO clones. However, differences were statistically significant for each KO clone compared to the same cells expressing EGFP-tagged, wildtype Cdc42 (t-test; p = 0.002). (**c**) Western Blotting of Cdc42 demonstrating complete absence in KO cell populations exemplified by KO clone #39.9 and reduced expression of this protein in Cdc42 flox/- cells compared to control fibroblasts. Tubulin was used as loading control.

Since we assumed these formins to be specific effectors of Cdc42 at the Golgi, we also explored Golgi architecture in fibroblastoid cells genetically deficient for Cdc42^36^. Two Cdc42 KO clones were analyzed, and could only be compared to the single parental cell line heterozygous for Cdc42 (flox/- cells) used as control (Fig. 4b). To our surprise, no significant differences were observed between KO cell lines and their respective control, perhaps due to the low gene dose as compared to control fibroblasts in the heterozygous control cell line that might already display phenotypic alterations in this respect (Fig. 4c). However, if both KO clones were transfected with EGFP-tagged, wildtype Cdc42, this then caused a strong increase in cell numbers harboring a compact Golgi (Fig. 4a,b). Interestingly, Golgi dispersal was also observed upon instantaneous Cdc42 inhibition by a small molecule inhibitor^37^. Together, we conclude that FMNL2 and -3 can influence Golgi architecture, likely operating downstream of Cdc42.

### FMNL2/3 KO alters formation, maturation and/or sorting of endosomes and lysosomes

**Figure 5:**
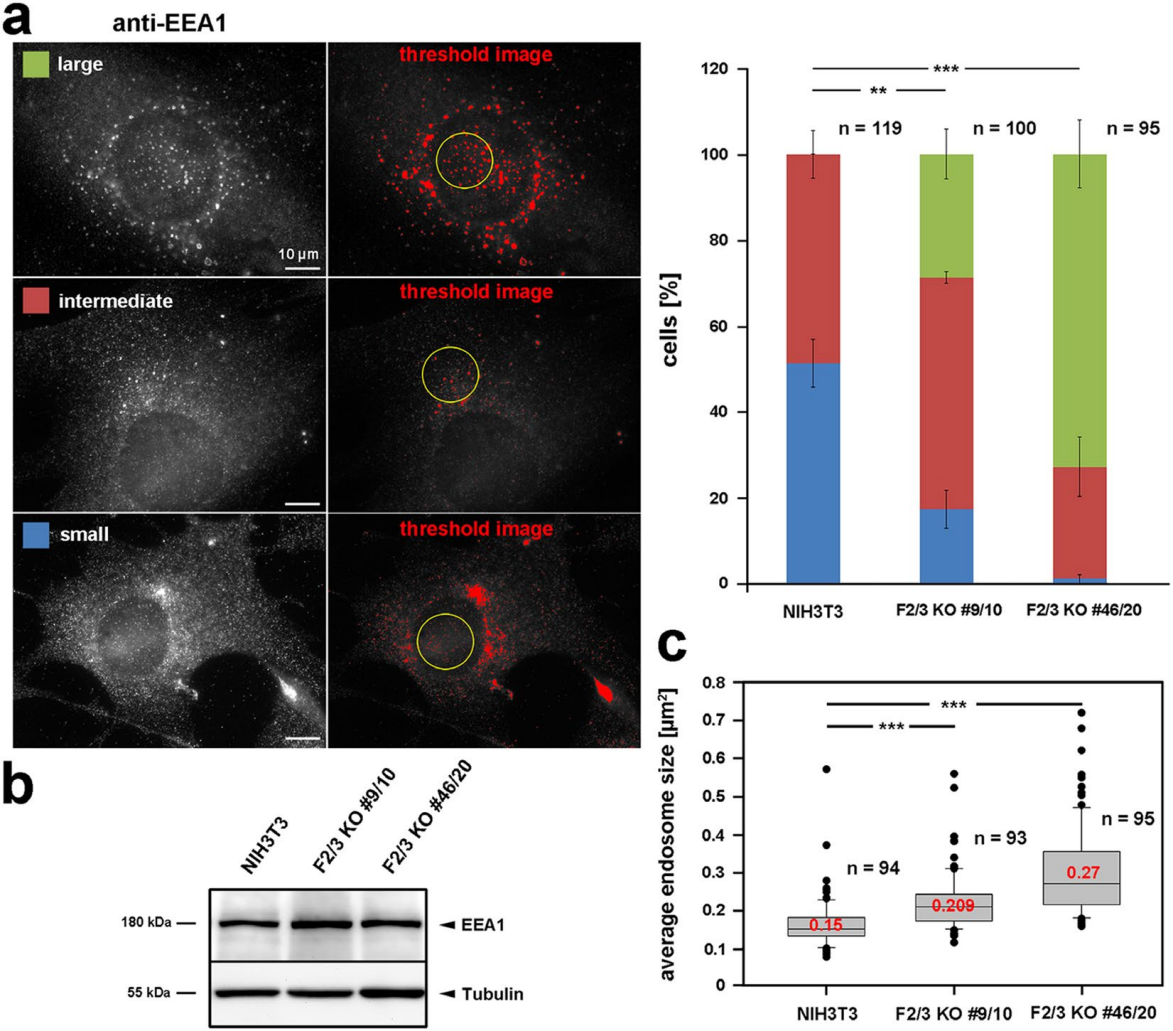
FMNL2/3 KO causes an accumulation of enlarged endosomes. (**a**) Classification of cells according to categories as depicted on the left (anti-EEA1 stainings), and corresponding examples of thresholded images used for computer-aided analyses (middle). Bar chart on the right summarizes results from manual categorizations of cells displaying respective endosome morphologies. Data are arithmetic means with error bars depicting sem, n gives numbers of cells analyzed. Statistical significance was determined for category “large endosome” (green, t-test). For quantifications of endosome sizes depicted in (**c**), individual, non-clustered endosomes in areas devoid of nonspecific background were included only, as exemplified by yellow circles in images in (**a**). Average endosome sizes were determined using the ImageJ particle analysis plugin. (**b**) Representative Western Blot of early endosomal antigen 1 (EEA1) levels in control and KO cell lysates, demonstrating expression levels of this protein not to be affected upon FMNL2/3 KO. Tubulin served as loading control. (**c**) Box and whiskers plots displaying results from computer-aided quantification of average endosome size (µm^2^). Median values are given in red, boxes include 50% (25–75%) and whiskers 80% (10–90%) of all measurements; outliers are shown as dots. n, number of cells analyzed from three independent experiments. Differences in average endosome size in NIH3T3 control and each FMNL2/3 KO clone were determined to be statistically significant using non-parametric Mann-Whitney rank sum test.

Cdc42 has previously been recognized as trafficking regulator and consequently been shown to operate in receptor-mediated endocytosis and macropinocytosis as well as in recycling and biosynthesis pathways, thus influencing polarized traffic^19^. In yeast, Cdc42 is known to perform this function through interaction with the formin Bni1^38, 39^. Concerning FMNL1γ, it was shown that its depletion causes an enlargement of lysosomes as well as redistribution of endosomes and mannose-6-phosphate receptors, implying regulatory functions in lysosomal and endosomal trafficking pathways^6^. Along these lines, FMNL2 was recently implicated in β1-integrin trafficking and reported to co-localize with the early and late endosomal markers Rab4/5 and Rab7, respectively^40^. Moreover, FMNL3 was also described recently to co-localize with cytoplasmic puncta of endocytic origin, although their nature was not further investigated^41^. All these data and aforementioned functions of Cdc42 prompted us to analyze potential effects of genetic FMNL2/3 disruption on endosome and lysosome patterns. Surprisingly, we found that a significant percentage of cells in both KO clones exhibited large endosomal structures positive for EEA1 staining, while none of the control cells were observed to display such a phenotype (Fig. 5a). In contrast, cells in which the anti-EEA1 staining was restricted to small structures (blue category in Fig. 5a), were significantly reduced in the absence of FMNL2 and -3, whereas cells containing a moderate amount of intermediate-sized endosomes (red category) remained relatively constant. Consistently, the observed changes caused a statistically significant increase in average endosome size in both KO cell lines (Fig. 5c). Of note, the observation cannot be explained by significant changes in total EEA1 levels (Fig. 5b).

**Figure 6:**
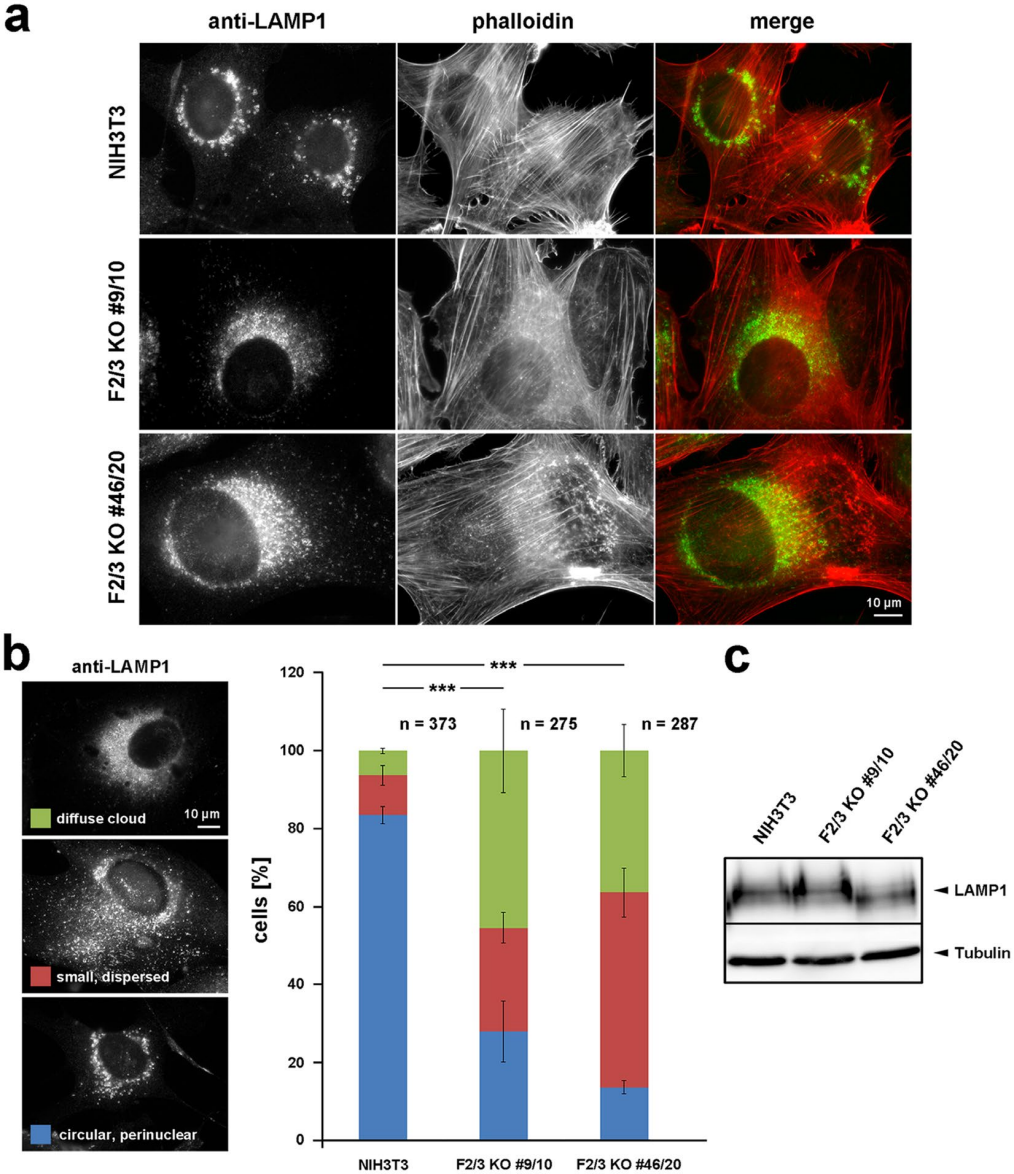
: FMNL2/3 KO causes malformation of lysosomes. (**a**) Anti-LAMP1 staining visualizing lysosomal compartments, with co-staining by phalloidin to reveal overall cell morphology; merges on the right, as indicated (actin filaments in red, lysosomes green). Note the prominent difference in appearance of lysosomes in control NIH3T3 cells versus FMNL2/3 KO clones. (**b**) Cell images on the left: Representative examples for manual categorization of cellular lysosome morphologies, as indicated (“circular perinuclear”, most representative for lysosome appearance in NIH3T3 control fibroblasts, “small dispersed” as intermediate phenotype and “diffuse cloud” as most severe phenotype. Right panel: Bar chart illustrating results of manual categorization according to the color code depicted on the left, with stacked columns corresponding to arithmetic means and error bars to sem; n gives number of cells analyzed, statistical significance of differences was determined for parameter “diffuse cloud” (t-test). (**c**) Western blot illustrating comparable expression levels of LAMP1 in control versus FMNL2/3 KO cells. Tubulin validates equal loading.

Molecules internalized by endocytosis are sorted into either recycling or degradation pathways using early endosomes. Actin inhibitors can impair endosomal trafficking^42^, causing fusion of early endosomes leading to formation of large endosomes containing molecules for both degradation and recycling^43^. Supplementary Movie 12 provides examples for how plasma membrane-associated FMNL formins can be endocytosed and accumulate on endo- or pinocytic spots arising from pinocytosed plasma membrane. It is tempting to speculate that elimination of FMNL2/3 and coincident alteration of actin dynamics at the plasma membrane might affect endo/pinocytosis and subsequent fusion- and/or sorting processes. Reminiscent of effects of FMNL1 knockdown on lysosome formation^6^ as well as KD of the actin regulator cortactin causing enlargement of lysosomes^44^, we found a striking phenotype concerning the distribution of LAMP1-positive lysosomes in FMNL2/3 KO cells (Fig. 6). While in 80% of control cells, lysosomes appeared as circular organelles in the perinuclear region, we found the majority of KO cells displaying a more peripheral distribution of lysosomes or an accumulation of lysosomes in diffuse, perinuclear clouds, in which typical, circular-shaped lysosomes were hardly visible (Fig. 6a,b). Again, overall expression levels of LAMP1 did not seem to be affected (Fig. 6c). However, in case of FMNL2/3 KO clone #46/20 (and in the B16-F1 FMNL2/3 KO clone #45/26; data not shown), the apparent molecular weight of LAMP1 in SDS-PAGE was changed, possibly due to modified protein glycosylation patterns, which, however, cannot fully explain observed phenotypes as it was not seen in FMNL2/3 KO #9/10 (Fig. 6c). In line with the observed malformation of lysosomes in 3T3, FMNL2/3 KO in B16-F1 cells caused changes in maturation of cathepsin D (Supplementary Fig. S19), which is known to require unimpaired lysosome function^45, 46^. Specifically, FMNL2/3 KOs exhibited significantly reduced levels of mature cathepsin D, whereas levels of immature cathepsin D appeared unaffected (Supplementary Fig. S19). Together, these data indicate that sorting, formation and/or maturation of lysosomes derived from either endosomes or Golgi are affected upon FMNL2/3 depletion.

Cdc42 is known to act at multiple levels of intracellular sorting processes, as missorting of basolateral proteins in epithelial cells upon functional deletion of Cdc42 does not only occur with newly synthesized proteins, but also with those recycled from the membrane^47^. Rab7 has previously been implicated in downstream endocytic trafficking, particularly in transport processes from early to late endosomes^48–50^. Several Rab proteins are known to function on early sorting and recycling endosomal compartments, whereas only Rab7 and Rab9 have been localized to late endosomes^51^. Rab7 regulates continuous fusion events between late endocytic structures and lysosomes. Since this protein constitutes a key regulator of lysosome biogenesis^52^, we stained control and FMNL2/3 KO cells for Rab7 and found it to display patterns remarkably similar to LAMP1-positive structures (Supplementary Fig. S20). Rab7/LAMP1 double-positive LE/Lys hybrid organelles appeared as circular, perinuclear vesicles in the majority of control cells. In contrast, circular Rab7-positive vesicular structures were only rarely found in FMNL2/3 KO cells, but rather showed up as diffuse and/or disperse staining similar to what was observed for LAMP1 (Supplementary Fig. S20). Together, all these data point to functions of FMNL family members in trafficking, likely involving both Golgi compartment and plasma membrane.

### FMNL2 and -3 regulate secretory, anterograde transport of VSV-glycoprotein

**Figure 7:**
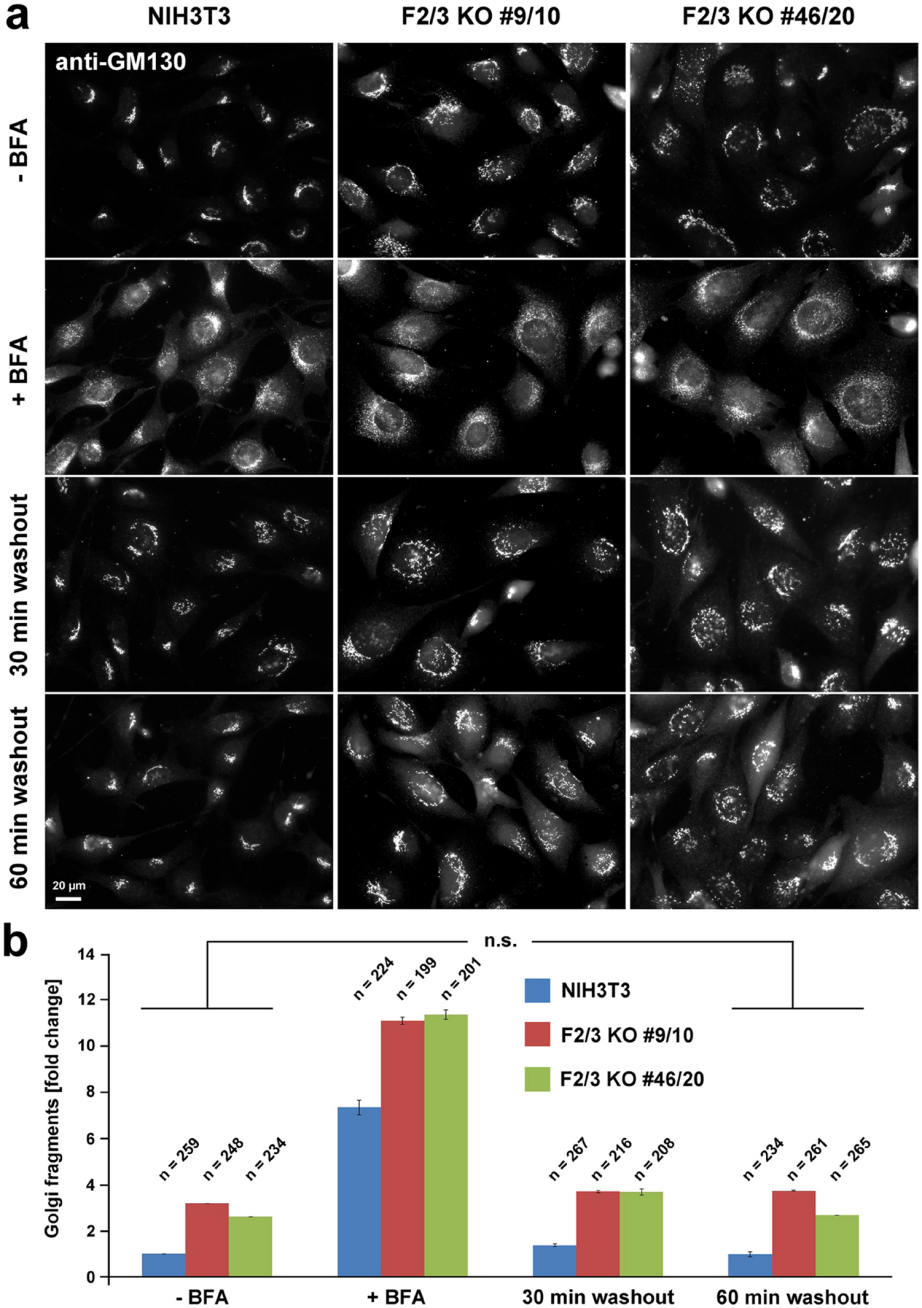
Loss of FMNL2/3 does not affect Golgi reassembly from the ER. (**a**) Representative GM130-stainings of NIH3T3 control and FMNL2/3 KO cell lines not treated (“−BFA”) or treated with Brefeldin A (2,5 µg/ml; “+BFA”) for 30 min, followed by replacement of BFA-containing medium with regular growth medium (“washout”) for the times indicated. Note that prior to BFA treatment, the Golgi apparatus is less organized and less compact in both KO cell lines compared to control cells, consistent with the data shown above. (**b**) Quantitation of average number of Golgi fragments in different cell lines and conditions as indicated. Both KO clones were normalized to the average number of Golgi fragments in control cells (“−BFA” = before treatment); n is number of cells analyzed. Differences between the 60 min washout stainings and those of untreated cells (“−BFA”) were confirmed not to be statistically significant (t-test) in all cell lines, indicating complete recovery from BFA-treatment, irrespective of the presence of FMNL subfamily formins.

To explore the role of FMNL2 and -3 in Golgi function in more detail, we first performed a brefeldin A (BFA) washout assay, constituting a standard assay for examining the assembly of the cellular Golgi compartment. 30 min treatment with BFA (2,5 µg/ml) disassembled the Golgi and caused its collapse back into the ER^53^. To explore potential defects in Golgi re-assembly, BFA was washed out and cells fixed at various time points after washout followed by staining for GM130 (Fig. 7). Interestingly, BFA caused a similar phenotype in both control and FMNL2/3 KO cells, with the Golgi appearing dramatically dispersed in both cases (Fig. 7a, “ + BFA”), but quantitatively more pronounced even in the KOs, as already seen without BFA (Fig. 7b). However, in both control as well as KO fibroblasts, the Golgi re-assembled to the state before BFA treatment with comparable kinetics. Taken together, these data indicate that FMNL2 and -3 are not involved in Golgi re-assembly from ER membranes upon BFA washout.

**Figure 8:**
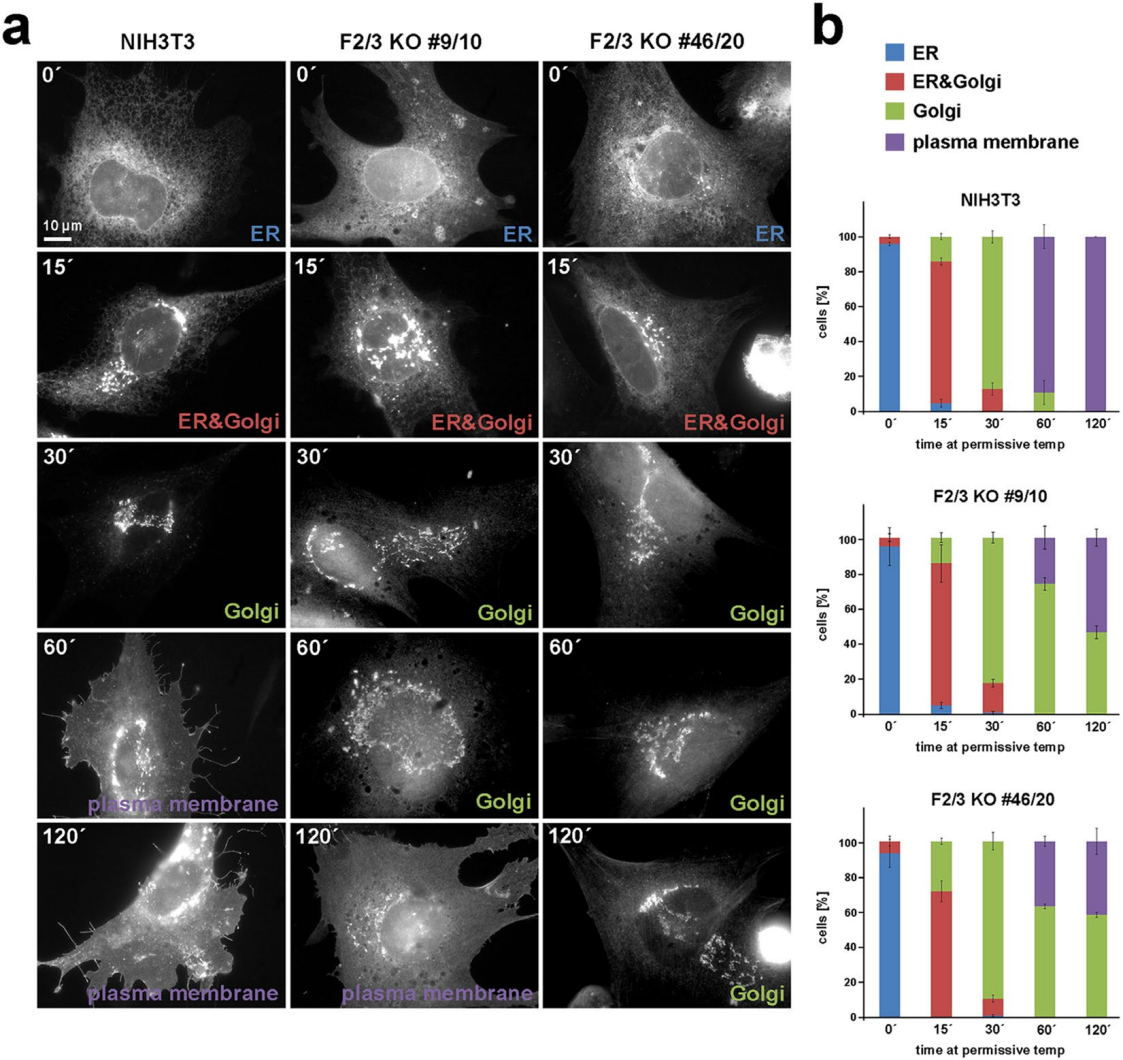
FMNL2/3 KO cells display defects in sorting and anterograde transport of VSV-G. (**a**) Representative examples of cells showing transport of ts045-VSV-G-EGFP from the ER to the plasma membrane as marked in each image. Time point 0′ indicates the starting point (ER retention) corresponding to the time point of shift to permissive temperature. (**b**) Summary of manual categorizations of localization patterns of ts045-VSV-G-EGFP in different conditions and cell lines as illustrated above. Data are from four independent experiments with more than 100 cells categorized for each time point. Error bars are sem. Note that in contrast to control cells, VSV-G-EGFP is still retained in the Golgi apparatus in a high fraction of KO cell populations at 60′ and 120′ time points. We also note that in KO cell lines only, a fraction of cells in each time point (of roughly 20% on average) displayed an unusual cytosolic and nuclear fluorescence, which was not characterized further and thus disconsidered for the manual categorization.

Next, we tested for potential functions of FMNL proteins in the anterograde, secretory pathway using vesicular stomatitis virus glycoprotein (VSV-G) transport as model system, which was described to be unimpaired in previous studies upon FMNL1 knockdown^6^. Temperature-sensitive, EGFP-tagged VSV-G (ts045-VSV-G-EGFP)^54, 55^ was incubated at the restrictive temperature (40 °C) overnight to induce its retention in the ER (Fig. 8a, 0 min), and followed on its journey through the secretory pathway upon shifting to the permissive temperature (32 °C). Both KO clones performed reasonably well within the initial 30 min after temperature shift (Fig. 8a,b), in line with the observation that Golgi re-assembly from ER membranes after BFA treatment was unaffected (Fig. 7). However, while almost all control cells showed plasma membrane (PM) association of VSV-G after 60 min, a high fraction of FMNL2/3 KO cells retained VSV-G protein in the Golgi complex (Fig. 8a,b), indicating a defect of anterograde vesicle trafficking from the Golgi apparatus. To confirm this, we also quantified the intensity of VSV-G-EGFP signal in the PM after 60 and 120 min incubation at permissive temperature (Supplementary Fig. S21). Only cells that showed clear PM incorporation at these time points were included in this analysis. We found that after 60 min at permissive temperature, measured VSV-G signal in the plasma membrane appeared on average slightly reduced in FMNL2/3 KO cells as compared to controls, although this was statistically insignificant. However, after 120 min, we detected a significant reduction in average intensity of VSV-G in the PM of FMNL2/3 double KO cells as compared to controls (Supplementary Fig. S21). Together, these data suggest a moderate but clear reduction of the effectivity of anterograde transport upon FMNL2/3 loss of function. Such a scenario would also be consistent with the proposal of Cdc42, the potential upstream regulator of FMNL formins in trafficking processes through the Golgi, promoting anterograde tubular transport and inhibiting retrograde vesicular transport by competing with retrograde cargoes for binding to coatomer^56, 57^. Future work will have to establish how precisely actin assembly affects anterograde transport, but we currently favor the idea that this is mediated by the described role of Cdc42 in stimulating membrane curvature^56^, which might require active actin filament assembly.

Recent work demonstrated that the Golgi is a rigid structure, requiring forces greater than 100 pN to induce its deformation, and that actin assembly can confer rigidity to the Golgi apparatus^58, 59^. As we recently established FMNL2/3 to assemble lamellipodial filaments contributing significantly to the force production by this structure during cell migration^21^, it is thinkable that FMNL-generated actin filaments might also serve mechanical functions at the Golgi and during trafficking processes. Moreover, as actin assembly during vesicle trafficking has previously been connected to Arp2/3 complex function and its activator and Cdc42 effector N-WASP^60–62^, future work will have to dissect precise connections between the latter actin assembly pathway and FMNL formin functions downstream of Cdc42.

In conclusion, our data clearly extend our understanding of the complexity of FMNL formin subfamily function in cells, in particular of the widely expressed FMNL2 and FMNL3 subfamily members, and clearly establish that their major activities are not restricted to lamellipodia or filopodia, but must now be extended to various intracellular membranes including the *trans*-medial Golgi or endo- and lysosomes.

## Electronic supplementary material

Supplementary Information

FMNL2-EGFP co-localizes with Cdc42 in the perinuclear region.

FMNL2-EGFP accumulates at Golgi structures.

FMNL3-EGFP accumulates at Golgi structures.

FMNL2-EGFP associates with intracellular vesicles.

FMNL3-EGFP accumulates at intracellular vesicular structures.

Morphology of the cis-Golgi in NIH control fibroblast, example 1.

Morphology of the cis-Golgi in NIH control fibroblast, example 2.

FMNL2/3 removal causes a fragmented Golgi morphology, clone #9/10, example 1.

FMNL2/3 removal causes a fragmented Golgi morphology, clone #9/10, example 2.

FMNL2/3 removal causes a fragmented Golgi morphology, clone #46/20, example 1.

FMNL2/3 removal causes a fragmented Golgi morphology, clone #46/20, example 2.

FMNL2-EGFP dynamics during lamellipodium protrusion and membrane ruffling.
