# Author Correction: FMNL2 and -3 regulate Golgi architecture and anterograde transport downstream of Cdc42

**DOI:** 10.1038/s41598-019-54630-z

**Published:** 2019-11-26

**Authors:** Frieda Kage, Anika Steffen, Adolf Ellinger, Carmen Ranftler, Christian Gehre, Cord Brakebusch, Margit Pavelka, Theresia Stradal, Klemens Rottner

**Affiliations:** 1 Division of Molecular Cell Biology, Zoological Institute, Technische Universität Braunschweig, Spielmannstrasse 7, 38106 Braunschweig, Germany; 2 Department of Cell Biology, Helmholtz Centre for Infection Research, Inhoffenstrasse 7, 38124 Braunschweig, Germany; 3 Center for Anatomy and Cell Biology, Medical University of Vienna, Schwarzspanierstraße 17, 1090 Vienna, Austria; 4 Biomedical Institute, BRIC, University of Copenhagen, DK-2200 Copenhagen, Denmark

Correction to: *Scientific Reports* 10.1038/s41598-017-09952-1, published online 29 August 2017

This Article contains an error in the Methods section under the subheading ‘DNA-constructs’.

“For EGFP-tagging, the full length sequence was amplified using forward primer 5′-GAGGAATTCATGGGCAATGCTGCCGG-3′ and reverse primer 5′-GAGGGATCCCTAGTGGTGGTGATGATGG-3′ harboring a stop codon.”

should read:

“For EGFP-tagging, the full length sequence was amplified using forward primer 5′-GAGGAATTCATGGGCAATGCTGCCGG-3′ and reverse primer 5′-GAGGGATCCCTACAGGGGCATTTCCTCGCC-3′ harboring a stop codon.”

In addition, this Article contains an error in the Results and Discussion section under the subheading ‘Cdc42-induced FMNL2/3 accumulation stimulates formin-specific actin filament assembly’.

“Importantly, respective FMNL2 and FMNL3 mutants (FMNL2-I1704A and FMNL3-1649A, respectively) still co-localized with Cdc42, but failed to induce the prominent actin accumulations frequently seen with wildtype FMNL2 or FMNL3 (Fig. 2b).”

should read:

“Importantly, respective FMNL2 and FMNL3 mutants (FMNL2-I704A and FMNL3-I649A, respectively) still co-localized with Cdc42, but failed to induce the prominent actin accumulations frequently seen with wildtype FMNL2 or FMNL3 (Fig. 2b).”

