# Supplementary material for: FMNL2 and -3 regulate Golgi architecture and anterograde transport downstream of Cdc42
Source: Sci Rep. 2017 Aug 29;7:9791. doi: 10.1038/s41598-017-09952-1 (PMC5575334; doi:10.1038/s41598-017-09952-1)
Supplement: Supplementary file 1 — Supplementary Information [file 41598_2017_9952_MOESM1_ESM.pdf]

SUPPLEMENTARY INFORMATION

FMNL2 and -3 regulate Golgi architecture and anterograde transport downstream of Cdc42

Frieda Kage[1,2], Anika Steffen[2], Adolf Ellinger[3], Carmen Ranftler[3], Christian Gehre[1,2], Cord Brakebusch[4], Margit Pavelka[3], Theresia Stradal[2], Klemens Rottner[1,2,*]

[1]Division of Molecular Cell Biology, Zoological Institute, Technische Universität Braunschweig, Spielmannstrasse 7, 38106 Braunschweig, Germany; [2]Department of Cell Biology, Helmholtz Centre for Infection Research, Inhoffenstrasse 7, 38124 Braunschweig, Germany; [3]Center for Anatomy and Cell Biology, Medical University of Vienna, Schwarzspanierstraße 17, 1090, Vienna, Austria; [4]Biomedical Institute, BRIC, University of Copenhagen, DK-2200 Copenhagen, Denmark

[*]To whom correspondence should be addressed:

Phone: +49 171 8195199

Fax: +49 0531 391 3222

Email: k.rottner@tu-braunschweig.de

SUPPLEMENTARY FIGURES AND LEGENDS

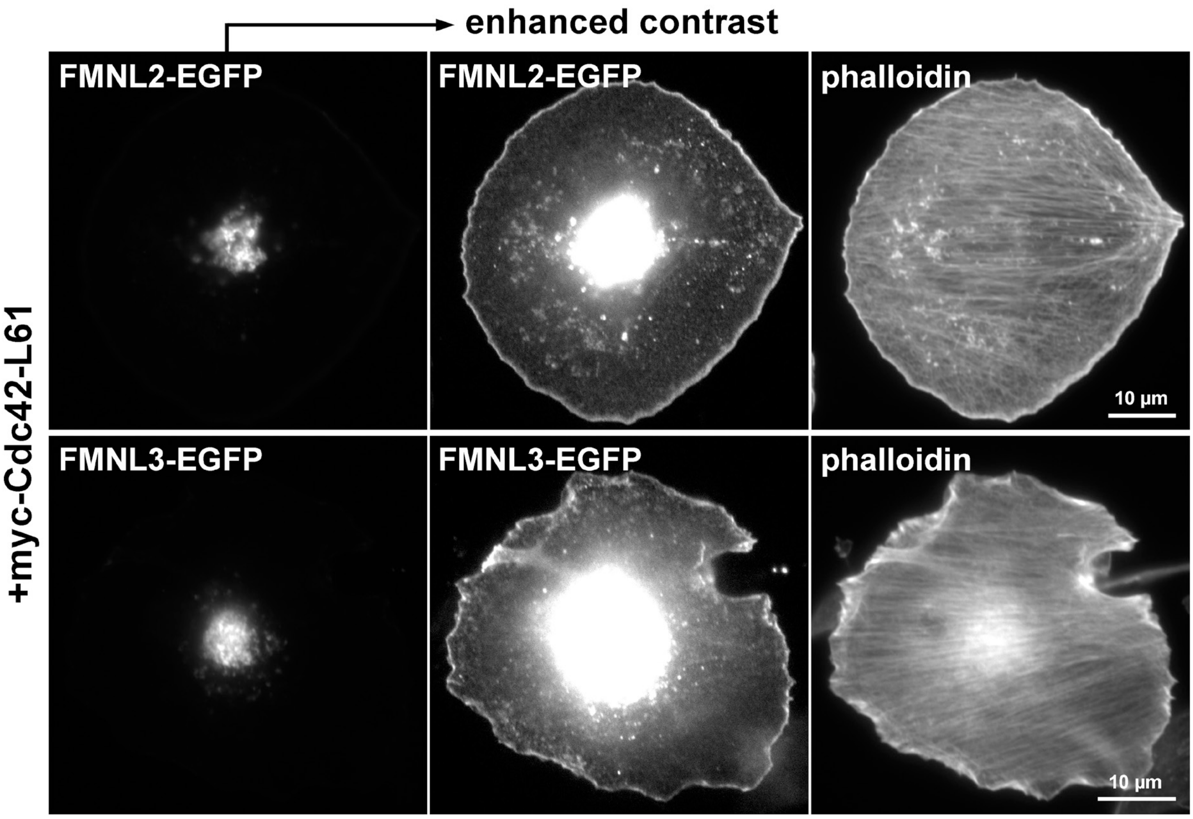

Figure S1: FMNL2 and -3 can localize to both Golgi and lamellipodia tips simultaneously, albeit at variable extents.

Epifluorescence images of B16-F1 cells co-expressing either FMNL2-EGFP or FMNL3-EGFP and myc-tagged Cdc42-L61. Cells were also labelled with fluorescent phalloidin (right panel). Pictures on the left demonstrate both formins to associate with the Golgi complex upon co-expression of active Cdc42-L61. However, enhanced contrast adjustment of the same images (leading to over-exposure at the Golgi) reveals both formins to simultaneously localize to lamellipodia tips (middle panel), as previously described[21,23], not apparent in these experimental conditions when normalizing to the strong accumulation at the Golgi apparatus (left panels).

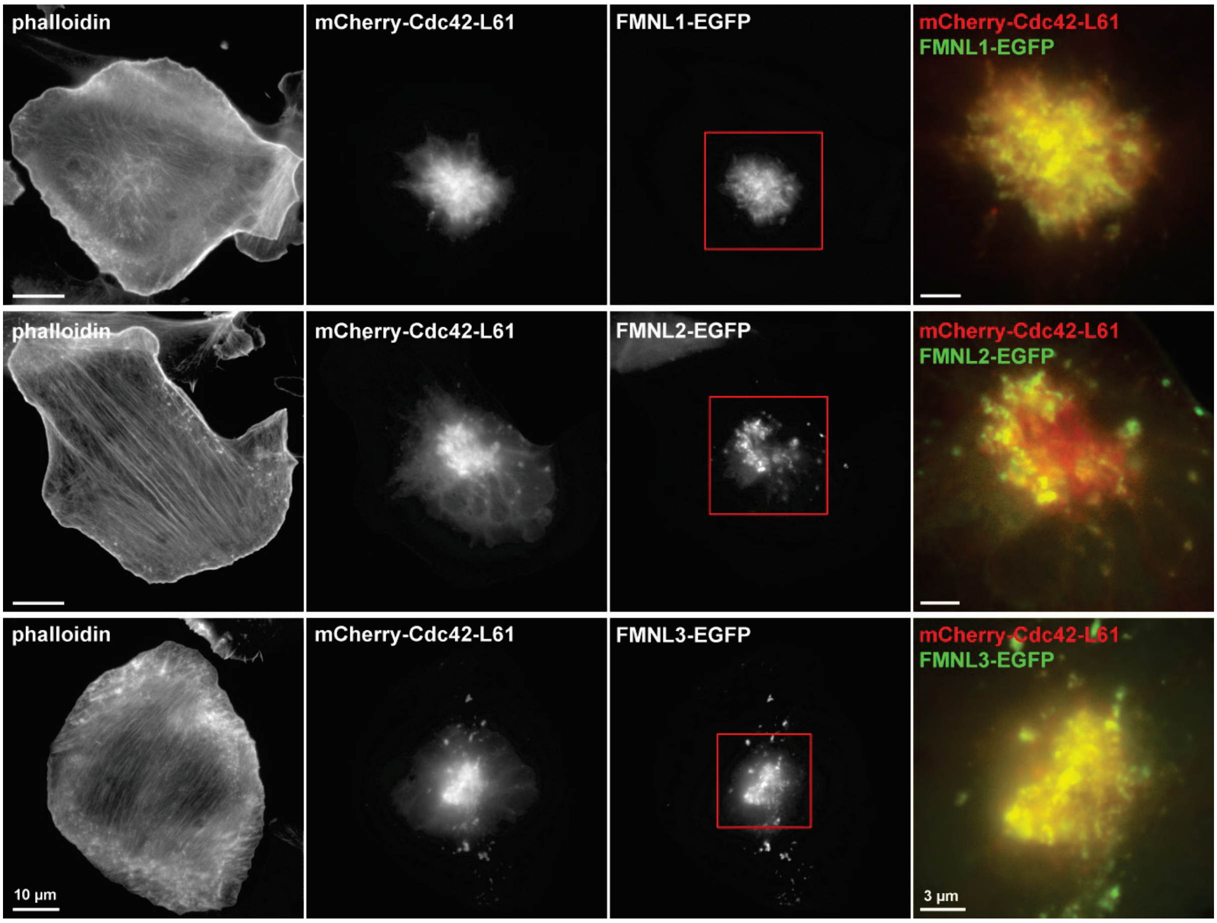

Figure S2: All FMNL subfamily members accumulate at the Golgi complex upon recruitment by active Cdc42.

B16-F1 melanoma cells transiently transfected with EGFP-tagged FMNL family member (green in merge on the right) and mCherry-tagged, constitutively active Cdc42 (green in merge) were fixed and counterstained for the actin cytoskeleton using blue phalloidin. Red insets are magnified in images shown in merges on the right (without phalloidin). Note robust co-localization of each FMNL family member with constitutively active Cdc42 (L61) at Golgi membranes and vesicular structures.

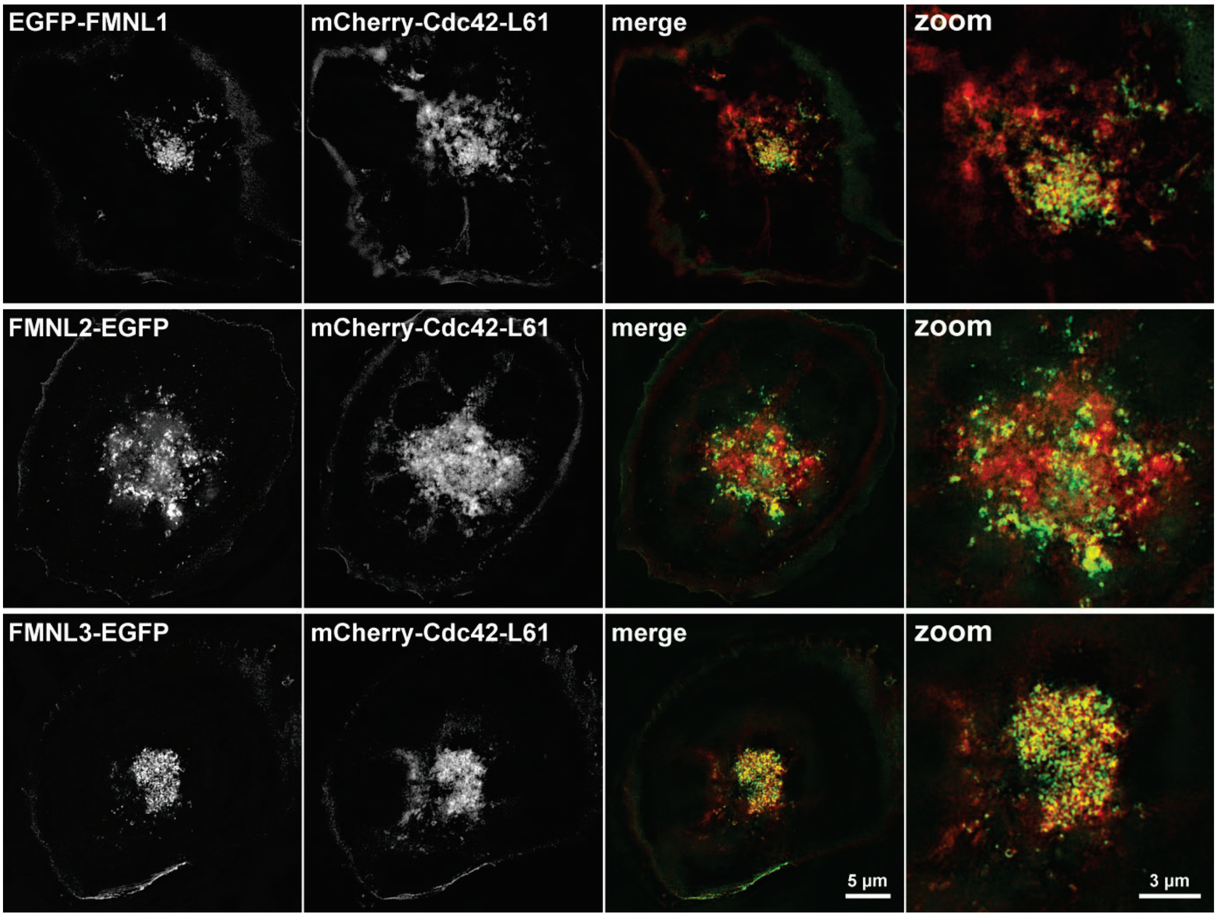

Figure S3: FMNL formins co-localize with Cdc42-L61 in the perinuclear region.

Superresolution images using 3D-Structured Illumination Microscopy (3D-SIM) of transfected B16-F1 cells, as indicated, revealing a clear perinuclear co-localization of all FMNL members with Golgi-resident mCherry-Cdc42-L61. Zoomed images on the right display merged color pictures (FMNL family member, green; mCherry-Cdc42-L61, red) of the perinuclear region in higher magnification (see also Supplementary Movie 1 for 3D-animation of FMNL2-EGFP data).

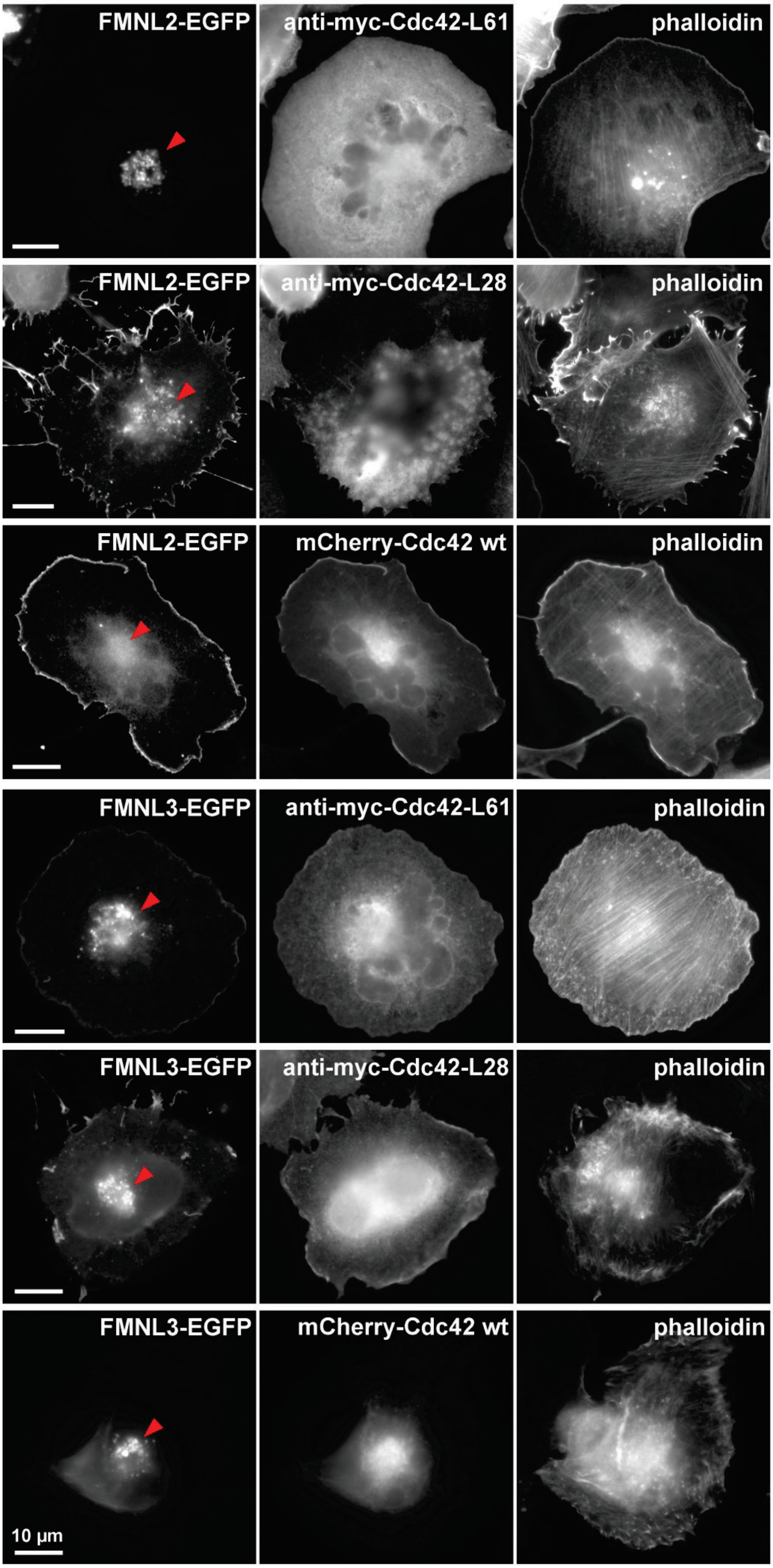
FMNL2-EGFP
anti-myc-Cdc42-L61
phalloidin
FMNL2-EGFP
anti-myc-Cdc42-L28
phalloidin
FMNL2-EGFP
mCherry-Cdc42 wt
phalloidin
FMNL3-EGFP
anti-myc-Cdc42-L61
phalloidin
FMNL3-EGFP
anti-myc-Cdc42-L28
phalloidin
FMNL3-EGFP
mCherry-Cdc42 wt
phalloidin
10 µm

Figure S4: Wildtype as well as a fast cycling mutant of Cdc42 (L28) promote Golgi accumulation of FMNL2 and FMNL3.

Epifluorescence images of B16-F1 cells transiently transfected with FMNL2- or FMNL3-EGFP in combination with constitutively active Cdc42-L61, the fast cycling mutant Cdc42-L28 or mCherry-tagged wildtype Cdc42, as stated in the pictures. Myc-tagged Cdc42 variants were visualized using anti-myc staining. Phalloidin was employed to stain for the F-actin cytoskeleton. Note that all Cdc42 constructs promoted recruitment of FMNL2 and -3 to the Golgi complex (red arrowheads), although to variable extents, with Cdc42-L61 being most and wildtype Cdc42 being least effective.

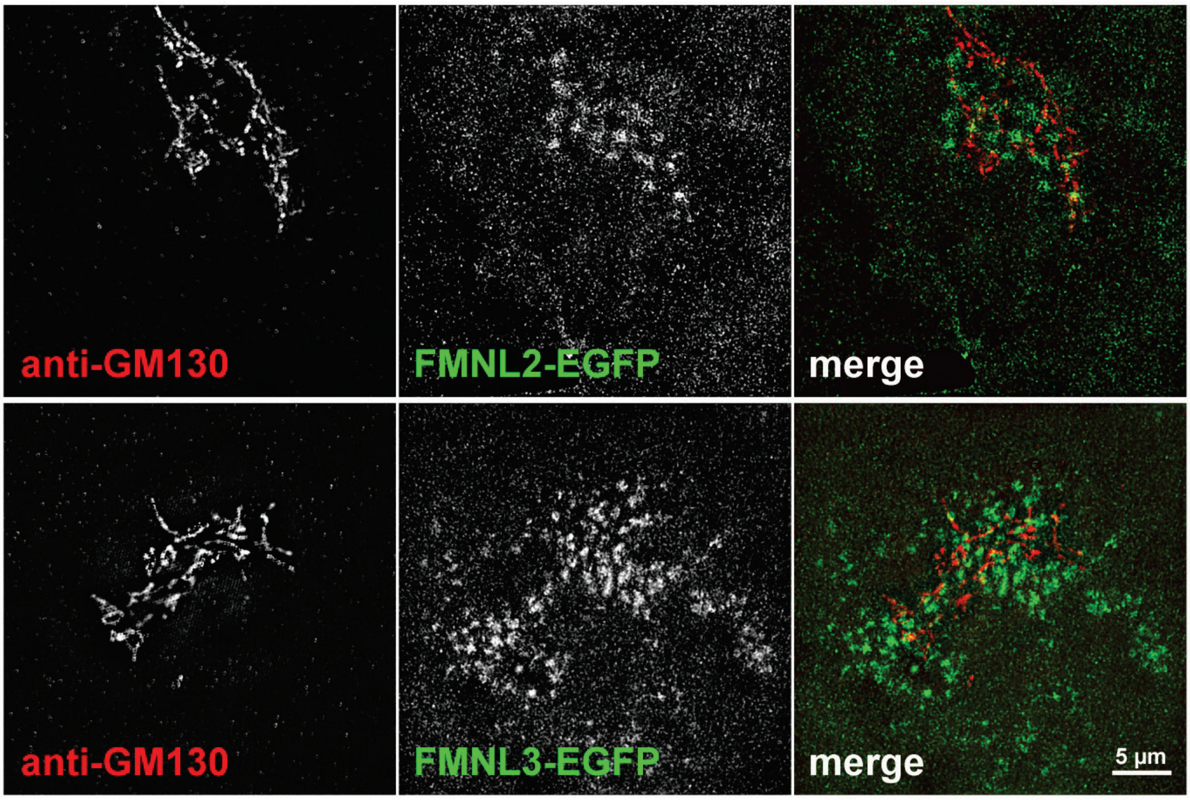

Figure S5: FMNL2 and -3 display perinuclear accumulation without co-expression of Cdc42.

Structured illumination microscopy superresolution images of COS-7 cells transiently transfected with either FMNL2- or FMNL3-EGFP, as indicated, and stained for the *cis*-Golgi compartment using a GM130-reactive antibody. Color-merged images on the right show an accumulation of these formins in the vicinity of *cis*-Golgi membranes, comparable to the localization data shown below.

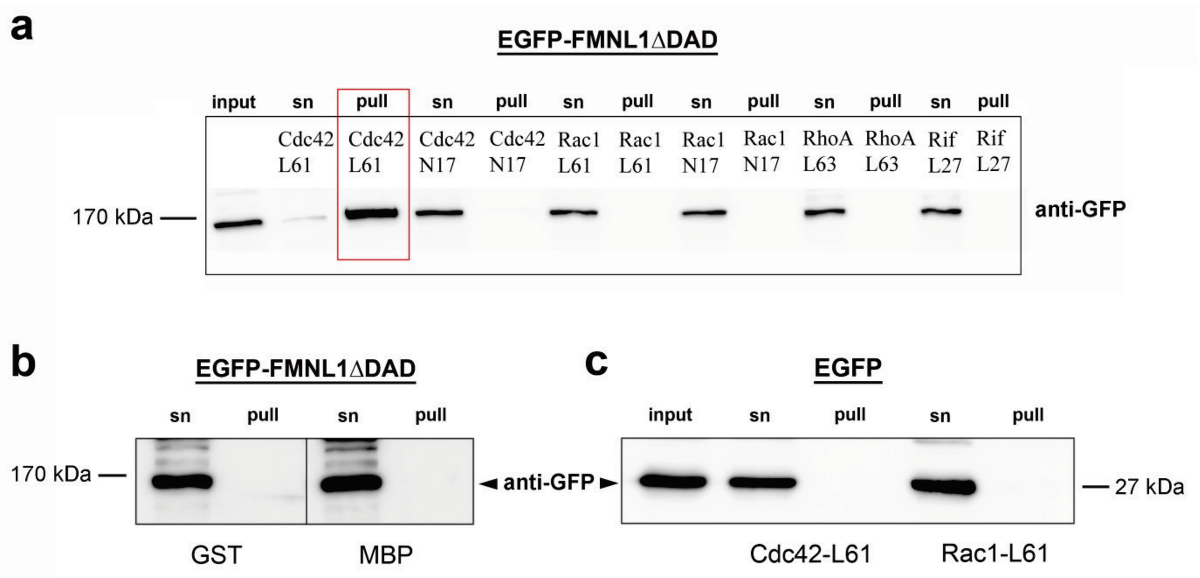

Figure S6: FMNL1 interacts with active Cdc42.

GST-pull down assay (using Rho-GTPases as indicated) of EGFP-tagged FMNL1ΔDAD (constitutively active version) ectopically expressed in B16-F1 cells and detected with anti-EGFP antibody, revealing binding of FMNL1 to active, GTP-bound Cdc42, but not dominant negative Cdc42 (N17) or active and inactive versions of Rac1, nor to active RhoA-L63 and Rif-L27 (the latter MBP-tagged).Red rectangle highlights specific pull down of EGFP-FMNL1ΔDAD with active, GST-tagged Cdc42 (L61). (**b**) Negative control showing that neither GST nor MBP alone was capable of pulling down EGFP-tagged FMNL1ΔDAD. (**c**) Negative control showing that neither constitutively active Cdc42 nor Rac1 was able to pull down EGFP alone.

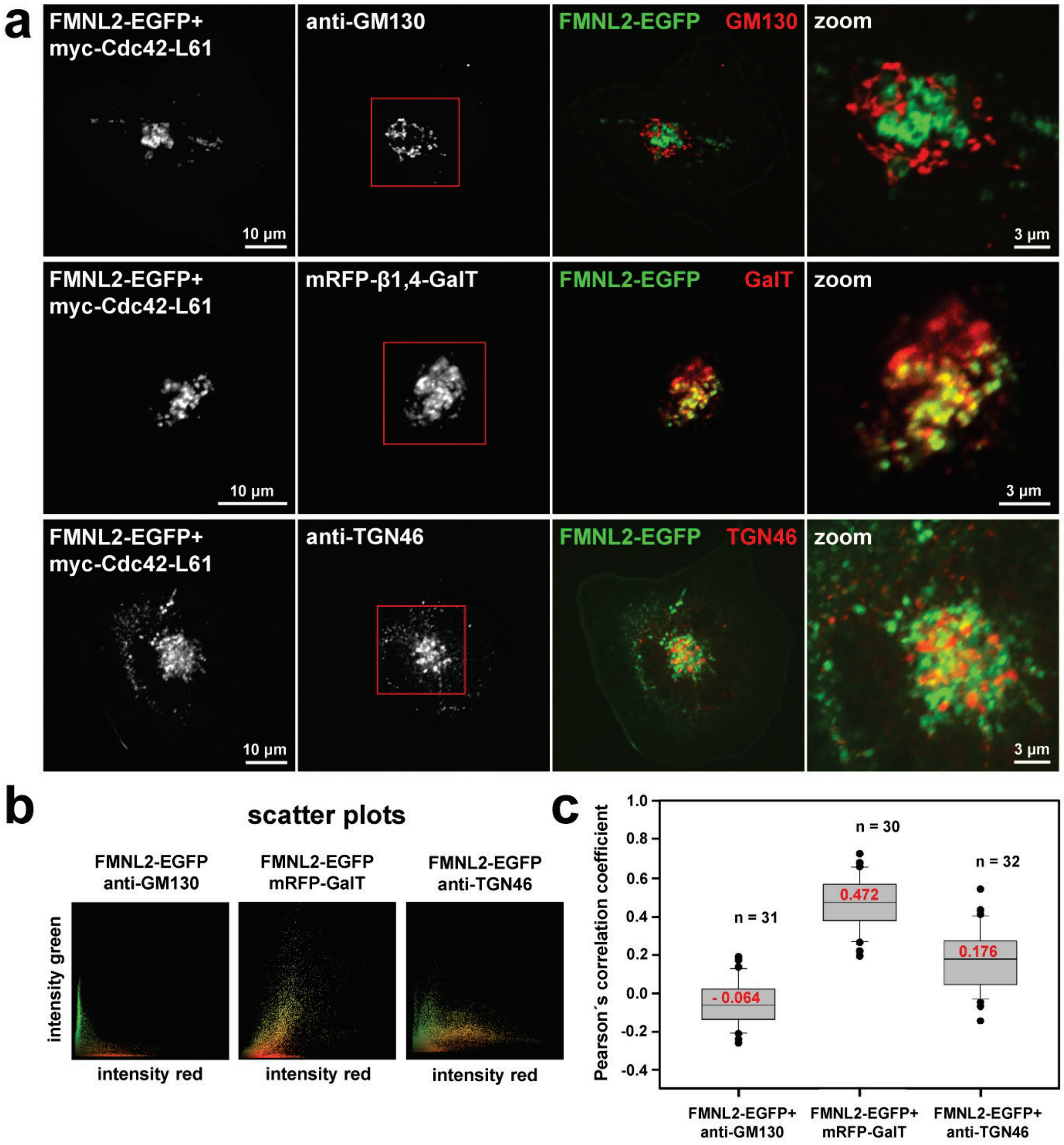

Figure S7: FMNL2 is specifically recruited to *trans*-medial Golgi cisternae.

(**a**) Spinning disc confocal fluorescence microscopy images of B16-F1 cells transiently co-transfected with FMNL2-EGFP and myc-tagged Cdc42-L61 were counterstained with anti-GM130 (Golgi matrix protein 130) and anti-TGN46 antibodies to define the *cis*-Golgi compartment (top) and TGN (bottom panels), respectively. For middle panels, FMNL2-EGFP and myc-tagged Cdc42-L61 were co-transfected with mRFP-β1,4-GalT, encoding a β-1,4-galactosyltransferase specifically accumulating in the *trans*-medial Golgi. Merged images display FMNL2-EGFP (green) and respective Golgi subcompartment (red) as indicated by labelling, and red rectangles mark insets magnified on the very right (zoom). (**b**) Representative scatter plots of perinuclear regions exemplify overlap quantities between proteins as specified. (**c**) Box and whiskers plots illustrating results from co-localization analysis, showing Pearson´s correlation coefficients for each of the stainings, as indicated. Boxes include 50% (25%-75%) and whiskers 80% (10%-90%) of all measurements, outliers are shown as

dots. Lines within boxes show medians, also given as numbers in red, n represents number of cells analyzed.

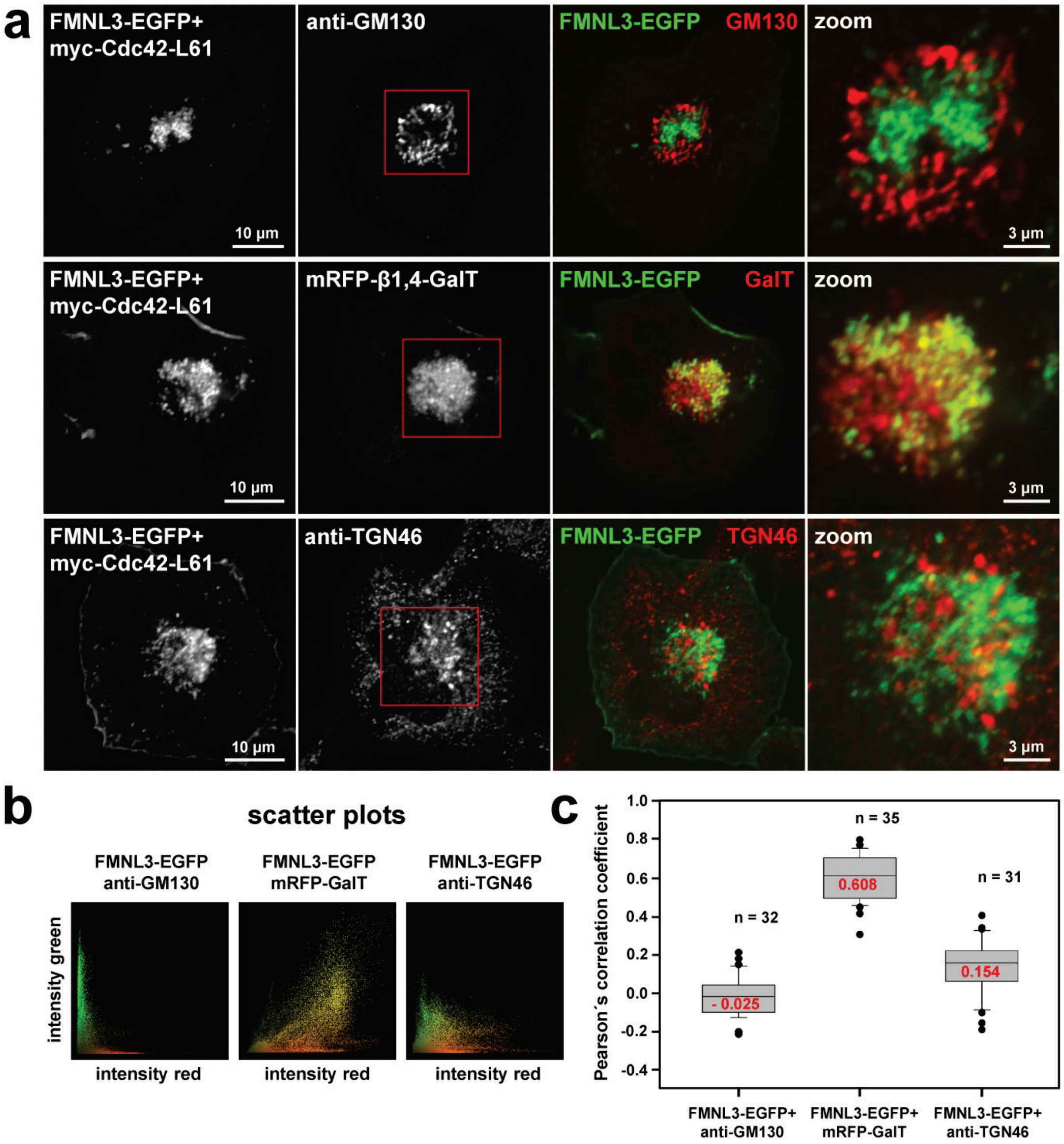

Figure S8: FMNL3 specifically associates with *trans*-medial Golgi cisternae.

(a) Spinning disc confocal fluorescence microscopy images of B16-F1 cells transiently co-transfected with FMNL3-EGFP and myc-tagged Cdc42-L61, as indicated, and counterstained with anti-GM130 and anti-TGN46 antibodies to mark *cis*-Golgi compartment (top) and TGN (bottom panels), respectively. For middle panels, FMNL3-EGFP and myc-tagged Cdc42-L61 were co-transfected with mRFP-β1,4-GalT to visualize the *trans*-medial Golgi. Merged images display FMNL3-EGFP (green) and respective Golgi subcompartment (red) as indicated by labelling, and red rectangles mark insets magnified on the very right (zoom). (**b**) Representative scatter plots of perinuclear regions exemplify overlap

quantities between proteins as specified. (**c**) Box and whiskers plots illustrating results from co-localization analysis, showing Pearson´s correlation coefficients for each of the stainings. Boxes include 50% (25%-75%) and whiskers 80% (10%-90%) of all measurements, outliers are shown as dots. Lines within boxes are medians, also given as numbers in red; n, number of cells analyzed.

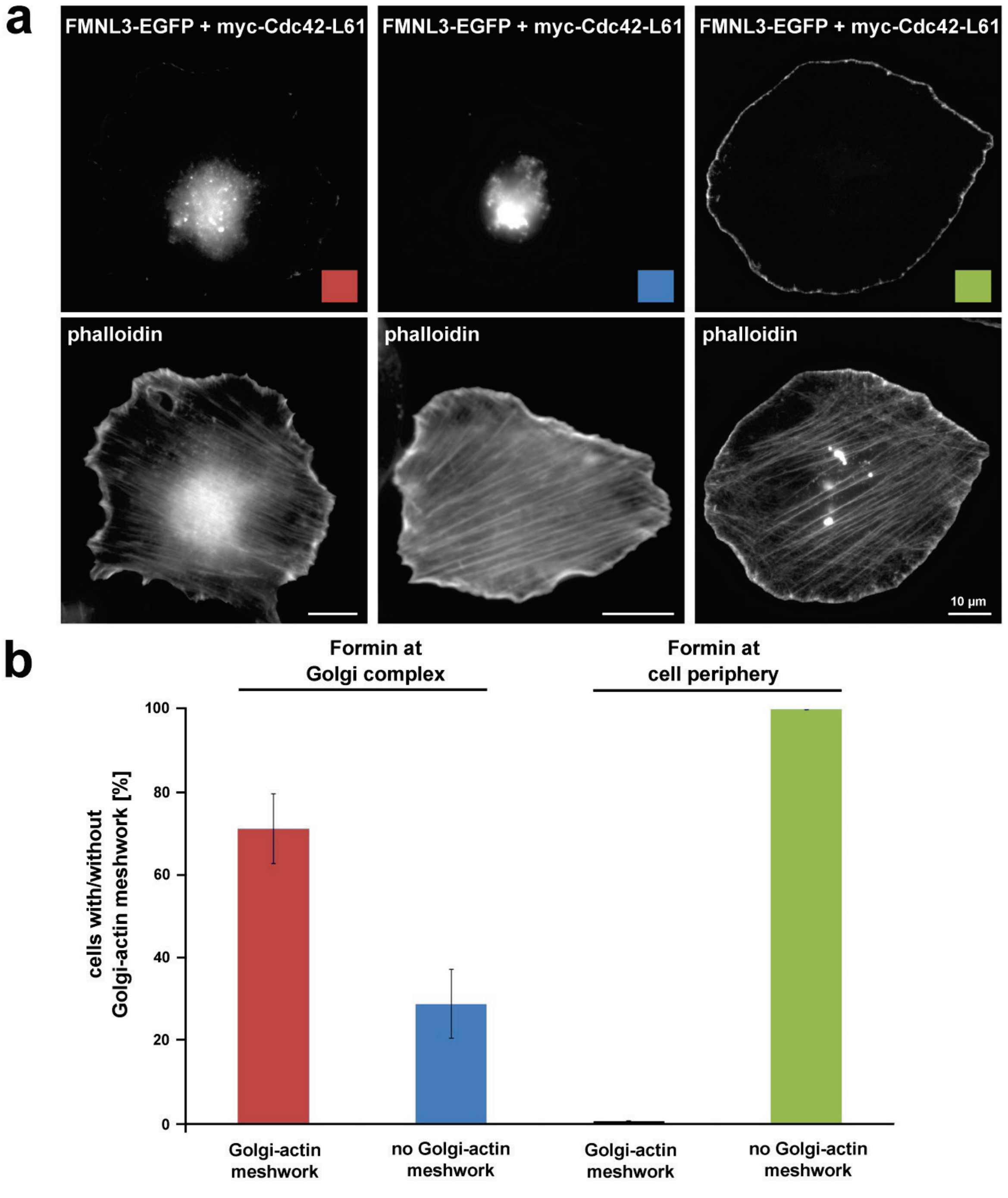

Figure S9: Golgi-located FMNL3 polymerizes F-actin at this organelle.

B16-F1 cells were co-transfected with FMNL3-EGFP and myc-Cdc42-L61, followed by fixation and phalloidin staining. (**a**) Representative examples of B16-F1 cells illustrating correlative cases of fomrin accumulation at Golgi *versus* cell periphery with actin accumulation at the Golgi. Colored rectangles illustrate distinct categories defined, with red and blue rectangles marking cells in which the formin is strongly located at the Golgi complex and harboring (red rectangle) or not (blue rectangle) phalloidin-stainable actin filament meshworks around Golgi. The third category shows cells in which the formin is not targeted to the Golgi, but remains exclusively localized at the lamellipodium tip (green rectangle). (**b**) Summary of incidences of actin accumulation at the Golgi in transfected cells,

categorized as shown in (a). Data are displayed as arithmetic means of percentages of transfected cells (± sem) harboring or not harboring discernible Golgi-actin, as indicated. Note that actin accumulation was never found in cells lacking FMNL3-EGFP; $n > 100$.

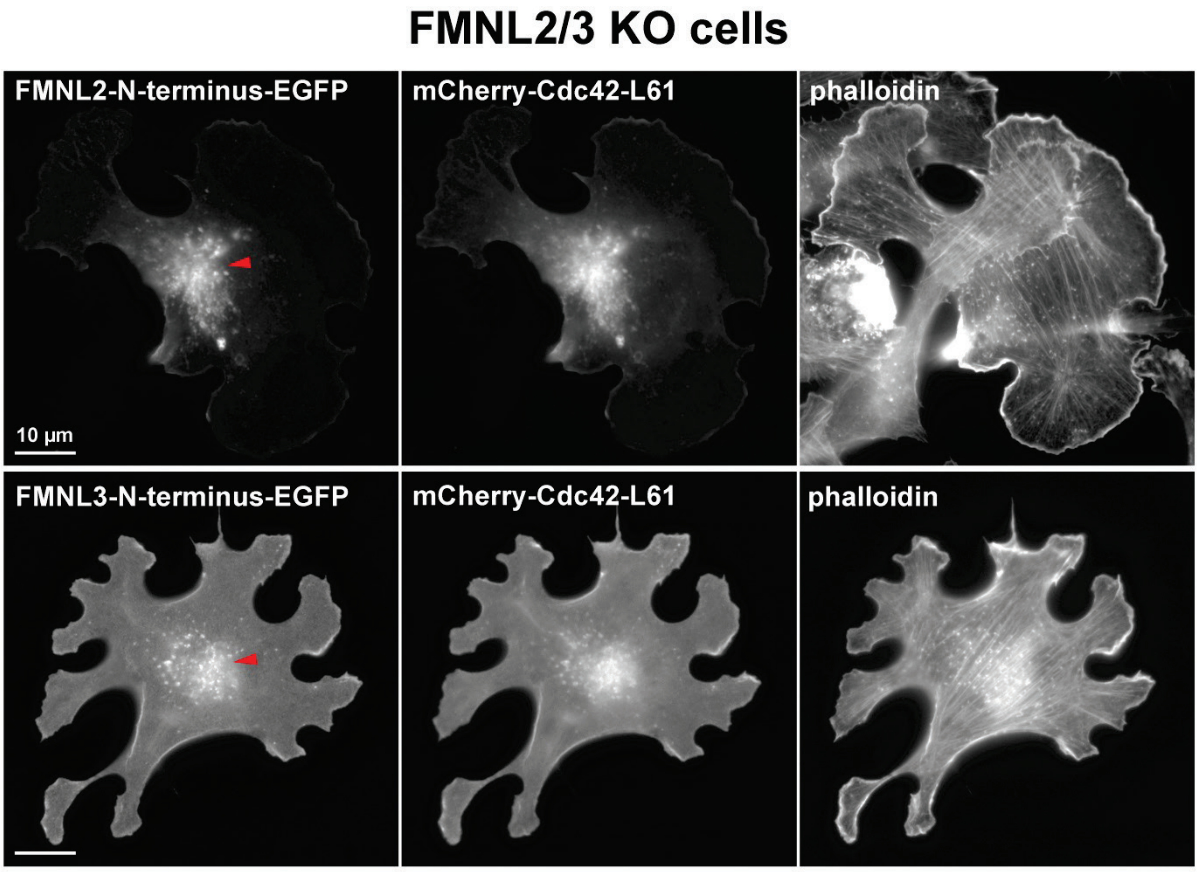

Figure S10: Cdc42 and N-terminal myristoylation are sufficient for Golgi targeting of FMNL2 and FMNL3.

FMNL2/3 KO B16-F1 cells were co-transfected with mCherry-tagged, constitutively active Cdc42 and the EGFP-tagged N-termini of FMNL2- or 3, both comprising respective N-terminal myristoylation sites and GTPase binding domains. Upon fixation, cells were counterstained with phalloidin, as indicated.

Note that expressed FMNL2/3 N-termini overlap with Cdc42 accumulation at plasma and intracellular membranes, including the Golgi (red arrowheads). Recruitment behavior was independent of endogenous, full length FMNLs, as transfections were carried out in the absence of endogenous proteins.

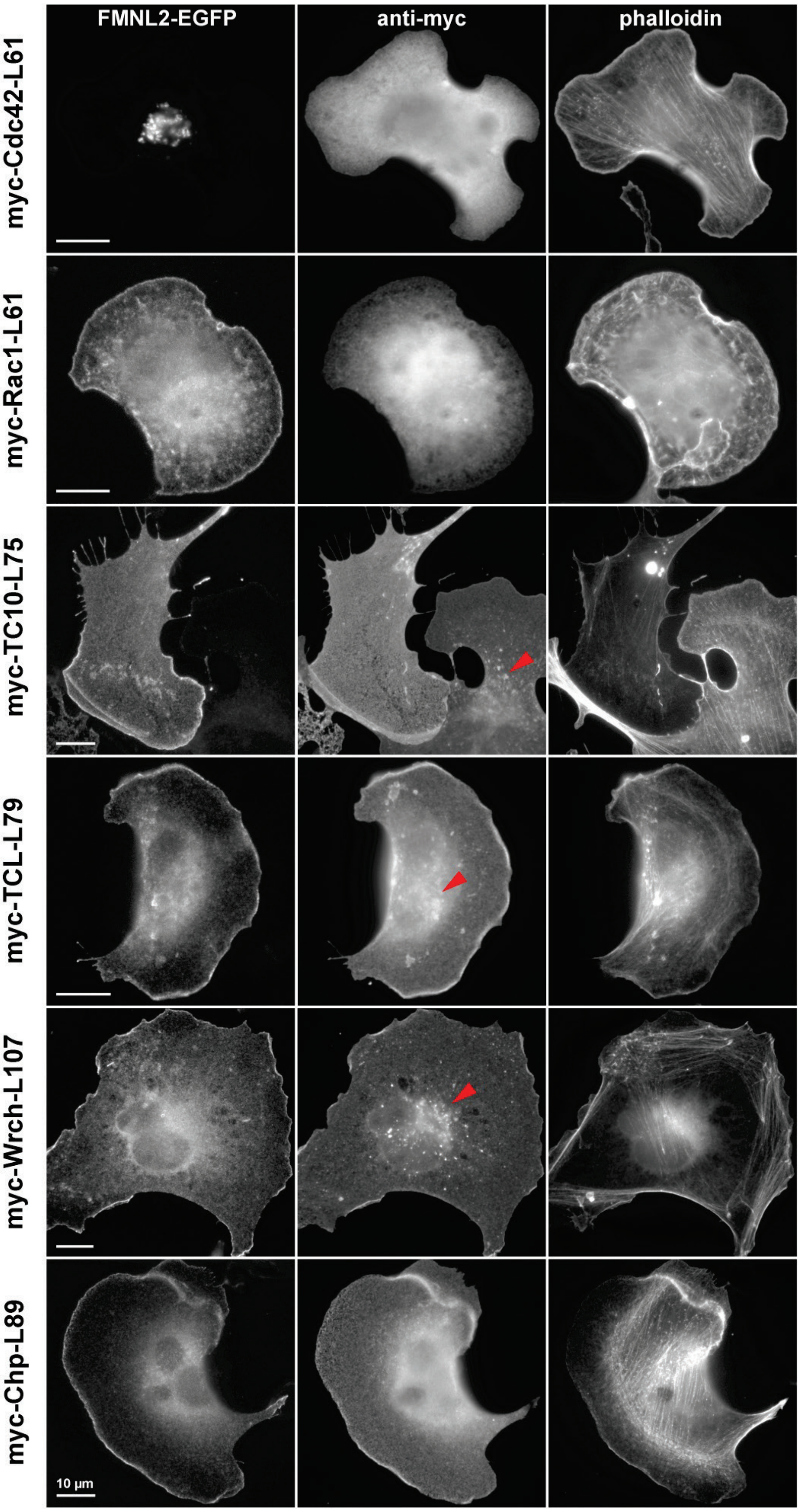

Figure S11: Golgi targeting of FMNL2 is solely stimulated by Cdc42.

Fixed and phalloidin-stained B16-F1 cells transiently co-transfected with FMNL2-EGFP plus constitutively active Rho-GTPases, as indicated. Expression of respective GTPase was validated by anti-myc staining. Cdc42 expression induced Golgi membrane association, as described above (top panel). However, none of the remaining Rho-GTPases tested here, including Cdc42-like GTPases TC10 and TCL, promoted FMNL2 recruitment to the Golgi apparatus. This was true even though the latter two and Wrch appeared to target to vesicular/Golgi-like structures, as expected[61,62] (red arrowheads).

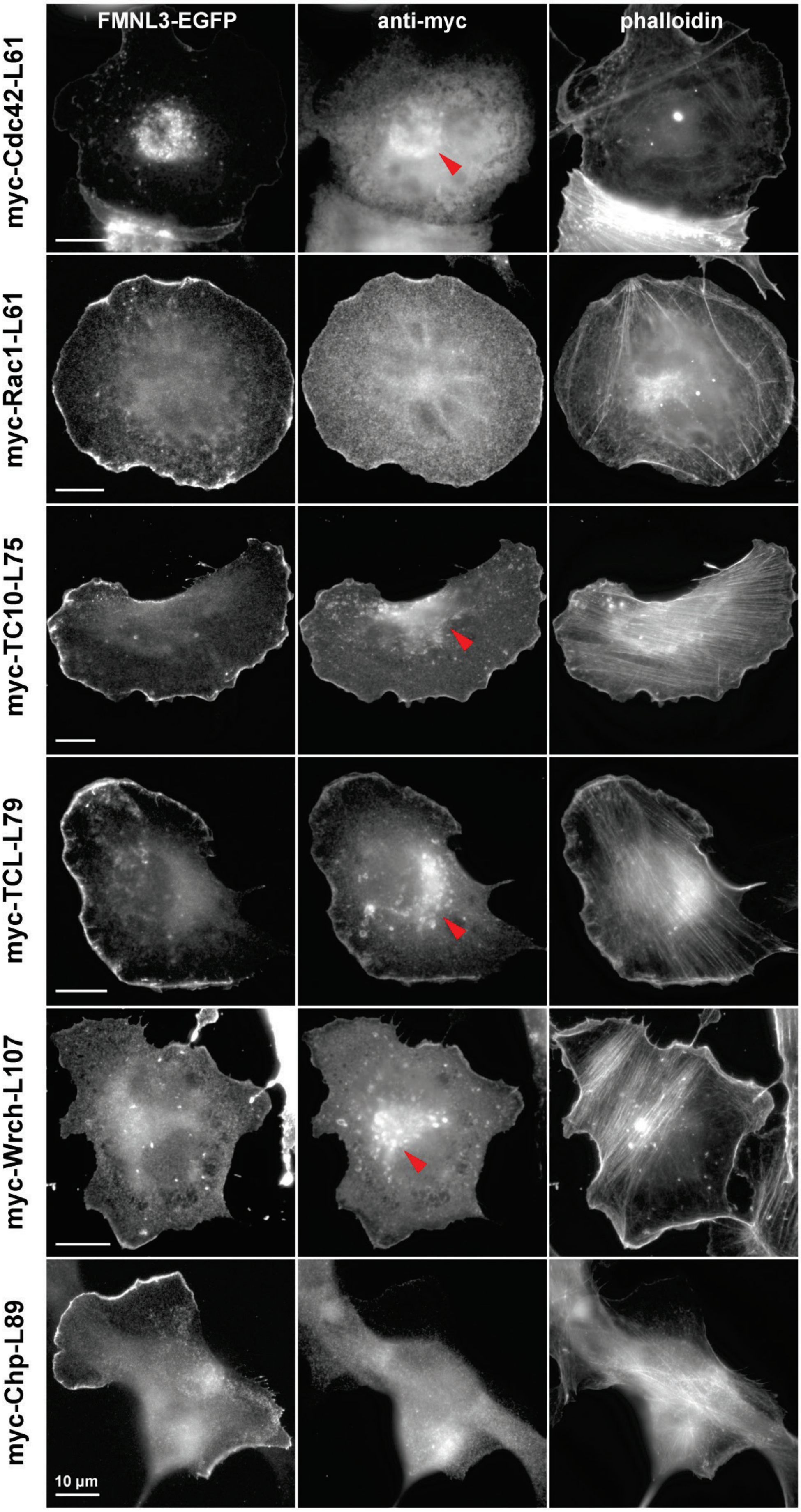
FMNL3-EGFP
anti-myc
phalloidin
myc-Cdc42-L61
myc-Rac1-L61
myc-TC10-L75
myc-TCL-L79
myc-Wrch-L107
myc-Chp-L89
10 µm

Figure S12: Golgi targeting of FMNL3 is exclusively stimulated by Cdc42.

B16-F1 cells were transiently co-transfected with FMNL3-EGFP and constitutively active Rho-GTPases, as indicated, and counterstained for the actin cytoskeleton with phalloidin. Expression of respective GTPase was validated by anti-myc staining. Cdc42 expression induced Golgi membrane association, as expected (top panel). As for FMNL2, none of the remaining Rho-GTPases tested here, including Cdc42-like GTPases TC10 and TCL, promoted FMNL3 recruitment to the Golgi apparatus. This was true in spite of TC10, TCL and Wrch all targeting to vesicular/Golgi-like structures, as expected[61,62] (red arrowheads).

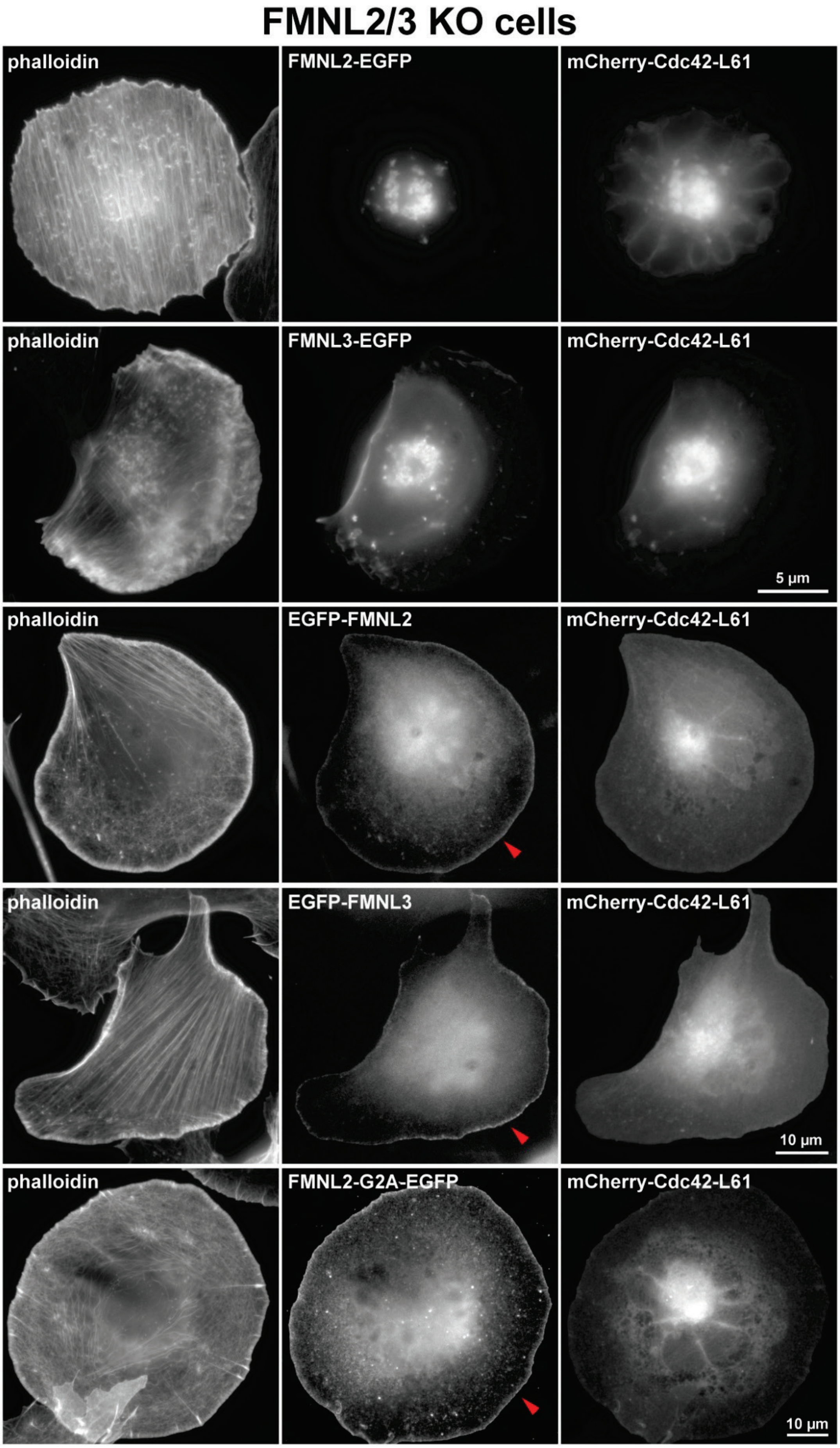
FMNL2/3 KO cells
phalloidin
FMNL2-EGFP
mCherry-Cdc42-L61
phalloidin
FMNL3-EGFP
mCherry-Cdc42-L61
5 µm
phalloidin
EGFP-FMNL2
mCherry-Cdc42-L61
phalloidin
EGFP-FMNL3
mCherry-Cdc42-L61
10 µm
phalloidin
FMNL2-G2A-EGFP
mCherry-Cdc42-L61
10 µm

Figure S13: N-terminal myristoylation is essential for Golgi membrane association of FMNL2 and -3.

FMNL2/3 KO cells were co-transfected with mCherry-Cdc42-L61 and either N- or C-terminally tagged FMNL2 or -3 or a non-myristoylatable point mutant of FMNL2 (FMNL2-G2A-EGFP), as indicated. N-terminal tagging was previously established to block N-terminal myristoylation[23], and is shown here to inhibit Golgi association of both FMNL2- and 3, in spite of the presence of co-transfected, constitutively active Cdc42. Blocking myristoylation of FMNL2 by mutation of residue 2 to alanine (FMNL2-G2A-EGFP) also eliminated Golgi association, in spite of EGFP fused to C-terminus of this variant, confirming that lack of myristoylation alone is sufficient for inhibiting Golgi accumulation.
Potential dimerization with endogenous FMNL formins was avoided through the use of FMNL2/3-deficient cells. Note that inhibition of N-terminal myristoylation did not block lamellipodial targeting of non-myristoylated FMNL variants (red arrowheads), consistent with previous data[21,23]. Note that C-terminally tagged FMNL2 and -3 can also target to the lamellipodium, but this is frequently masked in digital images displaying strong Golgi accumulation.

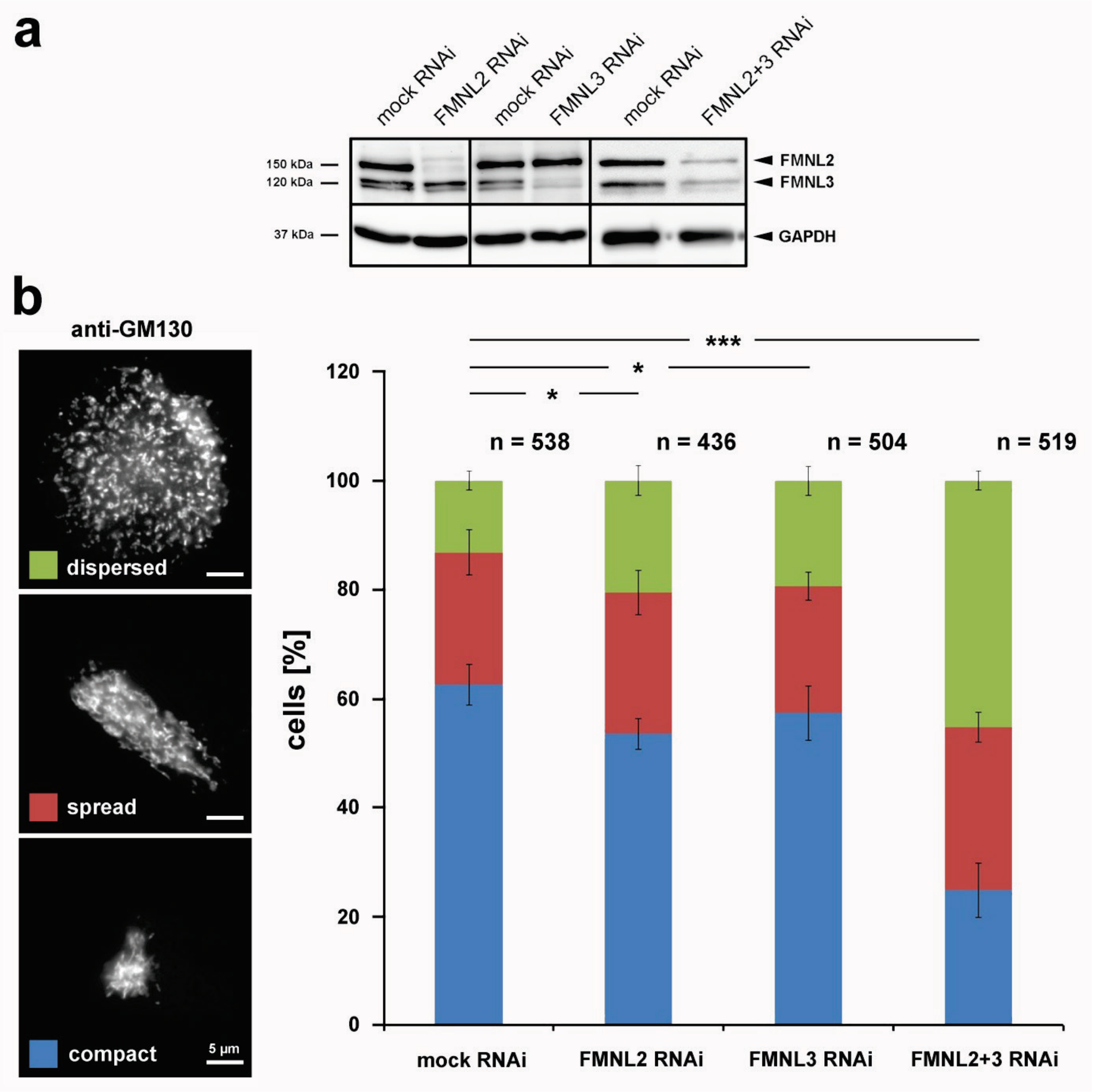

Figure S14: Combined knockdown of FMNL2 and -3 causes pronounced Golgi complex dispersal.

(**a**) Western Blot demonstrating efficiency of RNAi-mediated protein rundown using a FMNL2/3 reactive antibody and GAPDH as loading control. (**b**) Left panel: Representative images of compact (blue), spread (red) and dispersed (green) Golgi morphologies, exemplifying categories used for quantifications shown in bar graph on the right. GM130 was used as Golgi marker. Right panel: Bar graph summarizing quantitation of Golgi morphology categories in different experimental conditions, as indicated. Data are arithmetic means and standard errors of means; n equals number of cells analyzed; t-test confirmed statistically significant differences for category: “fragmented”. Although differences in individual FMNL2 or FMNL3 knockdown populations already appeared statistically significant, the changes were comparably modest. However, differences observed for double knockdown cells were strong and statistically highly significant, indicating overlapping functions for the two formins in promoting Golgi compaction.

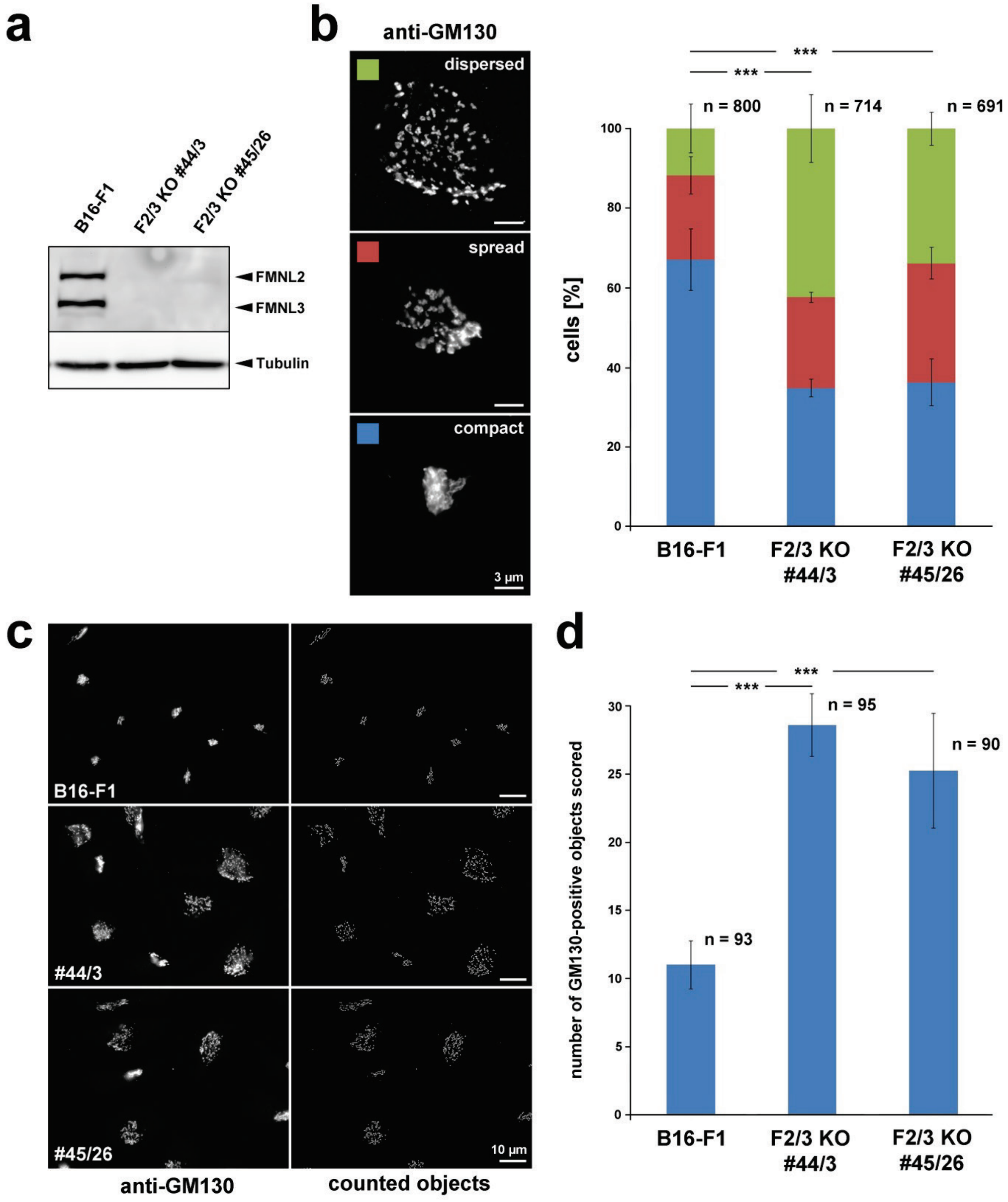

Figure S15: Genetic disruption of FMNL2 and -3 in B16-F1 cells causes Golgi dispersal.

(**a**) Western Blot revealing both CRISPR/Cas9-treated KO clones to be devoid of residual FMNL2 and -3 protein expression. Tubulin served as loading control. (**b**) Categorization of observed Golgi morphologies (left), and corresponding quantitation for the B16-F1 lines with genotypes as indicated. Data are displayed and statistics done as described for Supplementary Fig. S14b. (**c**) Representative examples of B16-F1 control and FMNL2/3 KO cells stained for GM130 (left) and outlines of objects identified and counted using Image J (right panel). (**d**) Bar chart showing average number of GM130-positive objects per cell determined using the ImageJ Squassh tool. Data are arithmetic means and

error bars standard errors of means (sem); n equals number of analyzed cells. Datasets were statistically compared using the non-parametric Mann-Whitney rank sum test.

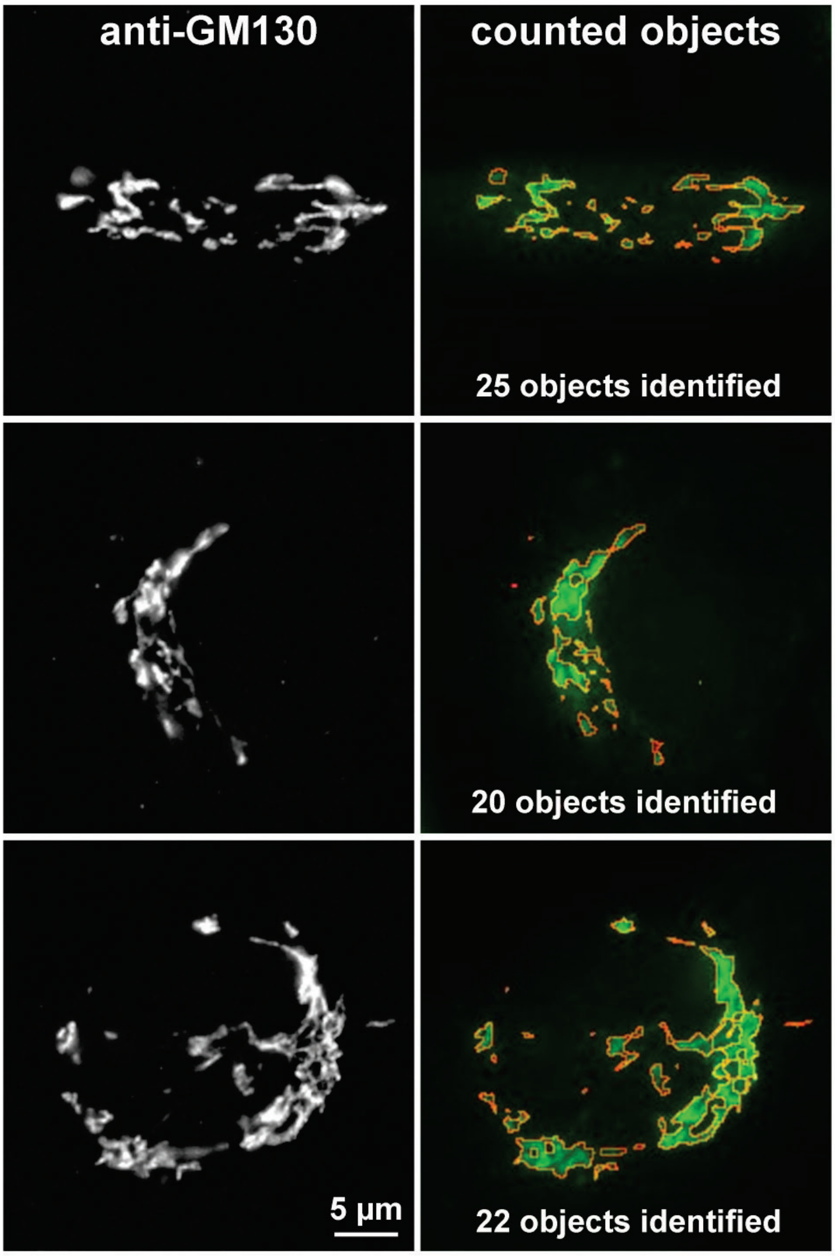

Figure S16: Squassh-mediated identification of GM130-stained objects.

Representative examples of NIH 3T3 fibroblasts stained for GM130 and photographed by widefield fluorescence microscopy (left panel). Right panel: Software-aided identification of separable objects (red boundaries) of GM130-staining (green), with numbers of quantified objects provided in each case.

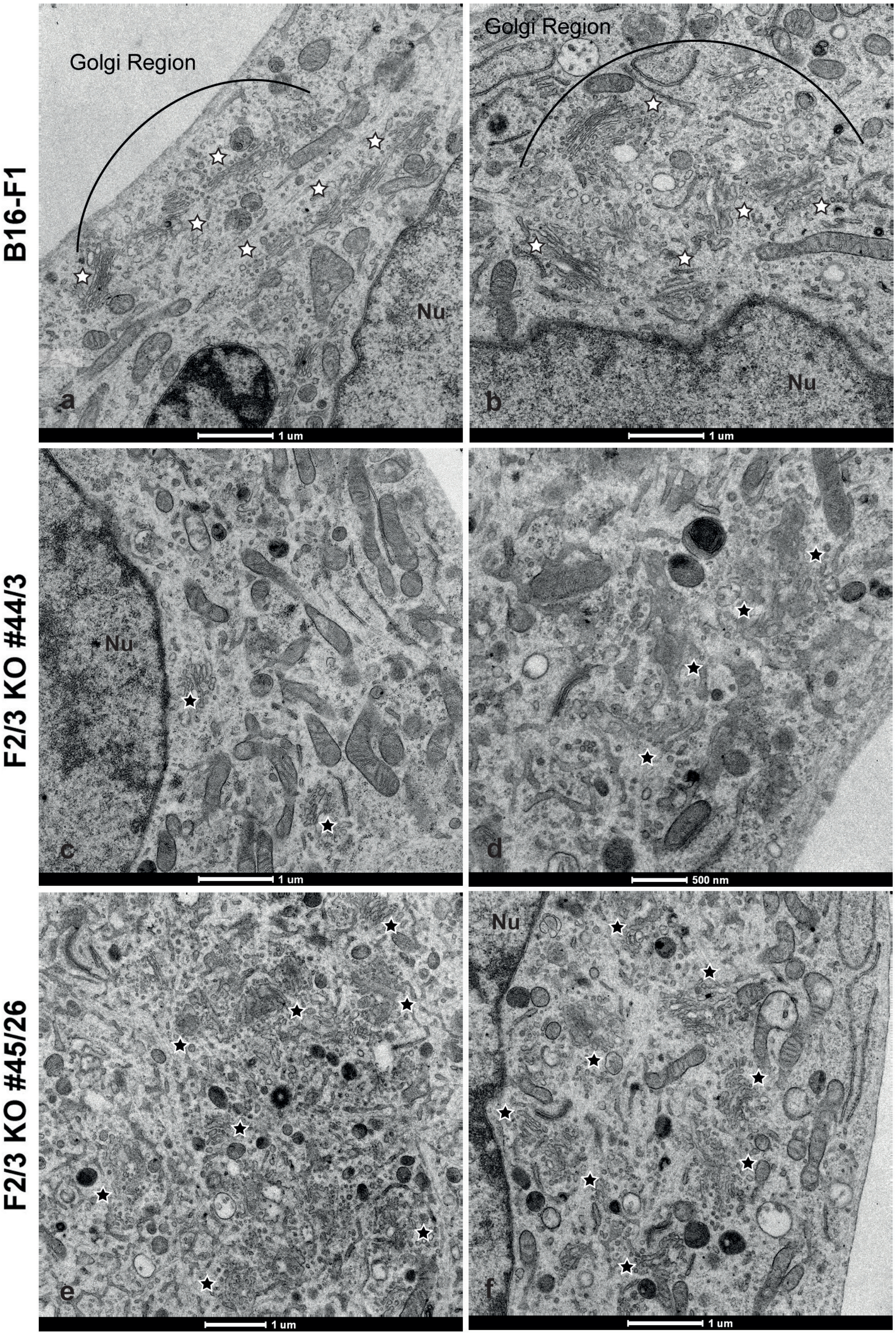
B16-F1
F2/3 KO #44/3
F2/3 KO #45/26
Golgi Region
Golgi Region
Nu
Nu
Nu
Nu
a
b
c
d
e
f
1 um
1 um
1 um
500 nm
1 um
1 um

Figure S17: Electron microscopy confirms disorganization of the Golgi complex in FMNL2/3 KO cells.

Electron micrographs showing Golgi apparatus morphologies, as they predominate in control B16-F1 cells (**a**, **b**) and FMNL2/3 KO cells (**c**, **d** and **e**, **f**). (**a**, **b**) Images depict the cytoplasm of control cells close to the nucleus with a distinct Golgi region composed of stacks of cisternae in a well ordered, parallel organization (white asterisks with black frames). (**c**, **d**) and (**e**, **f**) In FMNL2/3 KO cells, mini-stacks of short, loosely arranged cisternae and contorted membranes, surrounded by vesicles, are dispersed in the cytoplasm (black asterisks with white frames). More regularly organized stacks and disorganized mini-stacks can be seen located side by side.

Nu – nucleus; arches in panels (a) and (b) indicate the distinct Golgi regions apparent in the control cells.

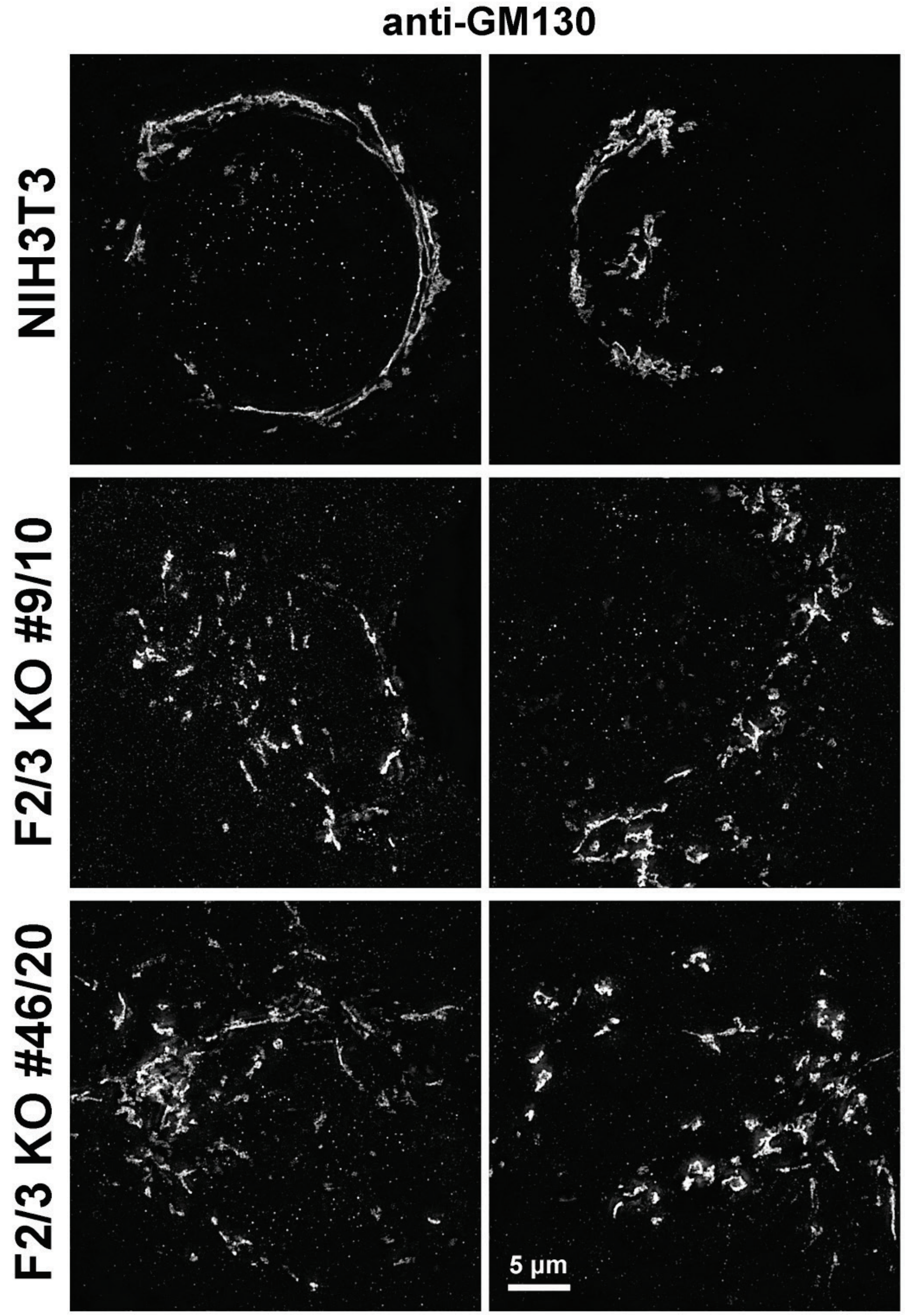

Figure S18: Genetic disruption of FMNL2 and -3 in NIH 3T3 fibroblasts causes Golgi dispersal and fragmentation.
Representative Structured Illumination Microscopy images of control NIH 3T3 and FMNL2/3 double KO cells stained for the Golgi apparatus using a GM130 marker. Two representative examples are shown for each cell line. Note that in case of both KO clones, the Golgi structure appears much more fragmented compared to control fibroblasts (see also Supplementary movies for 3D-reconstructions of the data).

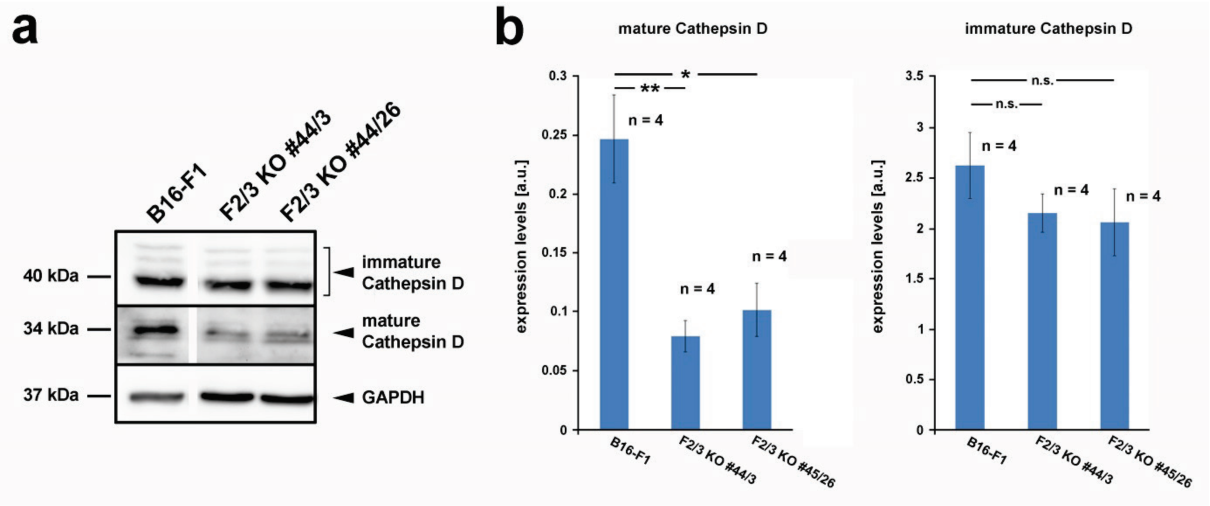

Figure S19: FMNL2/3 KO affects lysosomal maturation of Cathepsin D.

(**a**) Representative Western Blots showing levels of immature and mature Cathepsin D in FMNL2/3 double KO B16-F1 cell lines as compared to controls. Immature Cathepsin D (~52 kDa) is processed into an intermediate version (~ 40 kDa) within endo-lysosomal compartments followed by final cleavage into the mature form (~ 34 kDa). GAPDH was used as loading control. (**b**) Quantitation of Western Blot results represented as bar graphs for either mature Cathepsin D (left) or immature Cathepsin D (right). Expression levels are shown in arbitrary units [a.u.]; n represents the number of independently generated cell lysates. Expression levels of immature Cathepsin D were found not to be significantly different in both FMNL2/3 KO clones compared to controls, while differences in expression levels of mature Cathepsin D were determined to be significant (t-test).

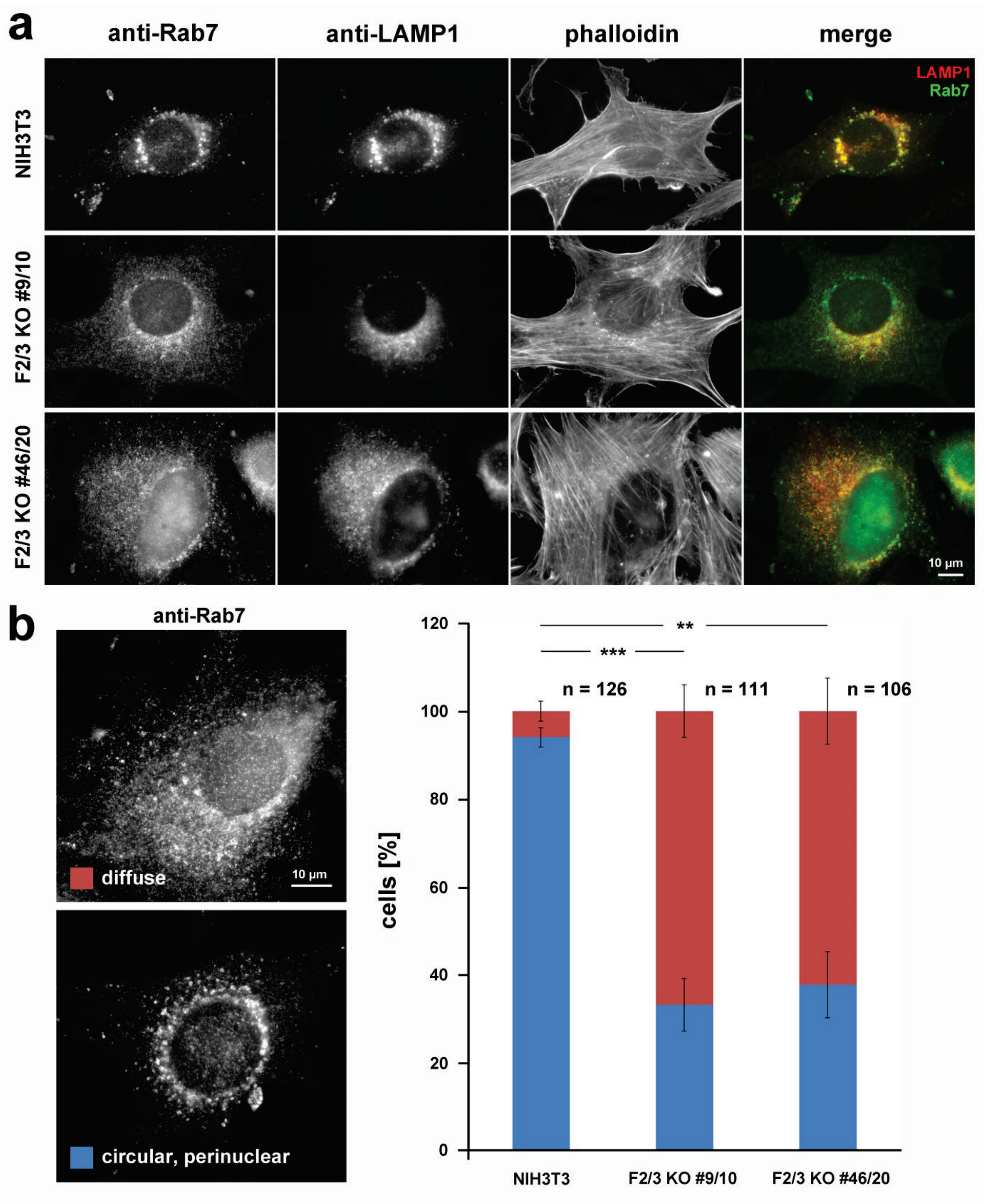

Figure S20: Late endosomal marker Rab7 is mislocalized upon loss of FMNL2/3.

(**a**) Representative examples of Rab7 (LE marker)/LAMP1 (lysosomal marker) and phalloidin triple-stainings of control NIH3T3 and FMNL2/3 KO clones, as indicated. Merges of Rab7 (green) and LAMP1 (red) are shown on the right. Note significant co-localization of both markers and a more diffuse, less confined staining of Rab7 and LAMP1 in case of FMNL2/3 KO cells. (**b**) Categorization of Rab7 stainings (let panel) and corresponding quantification depicted on the right; n corresponds to examined cells; dara are arithmetic means ± sem. Differences in Rab7 categorization as explored for parameter “diffuse” were determined to be statistically significant using t-test.

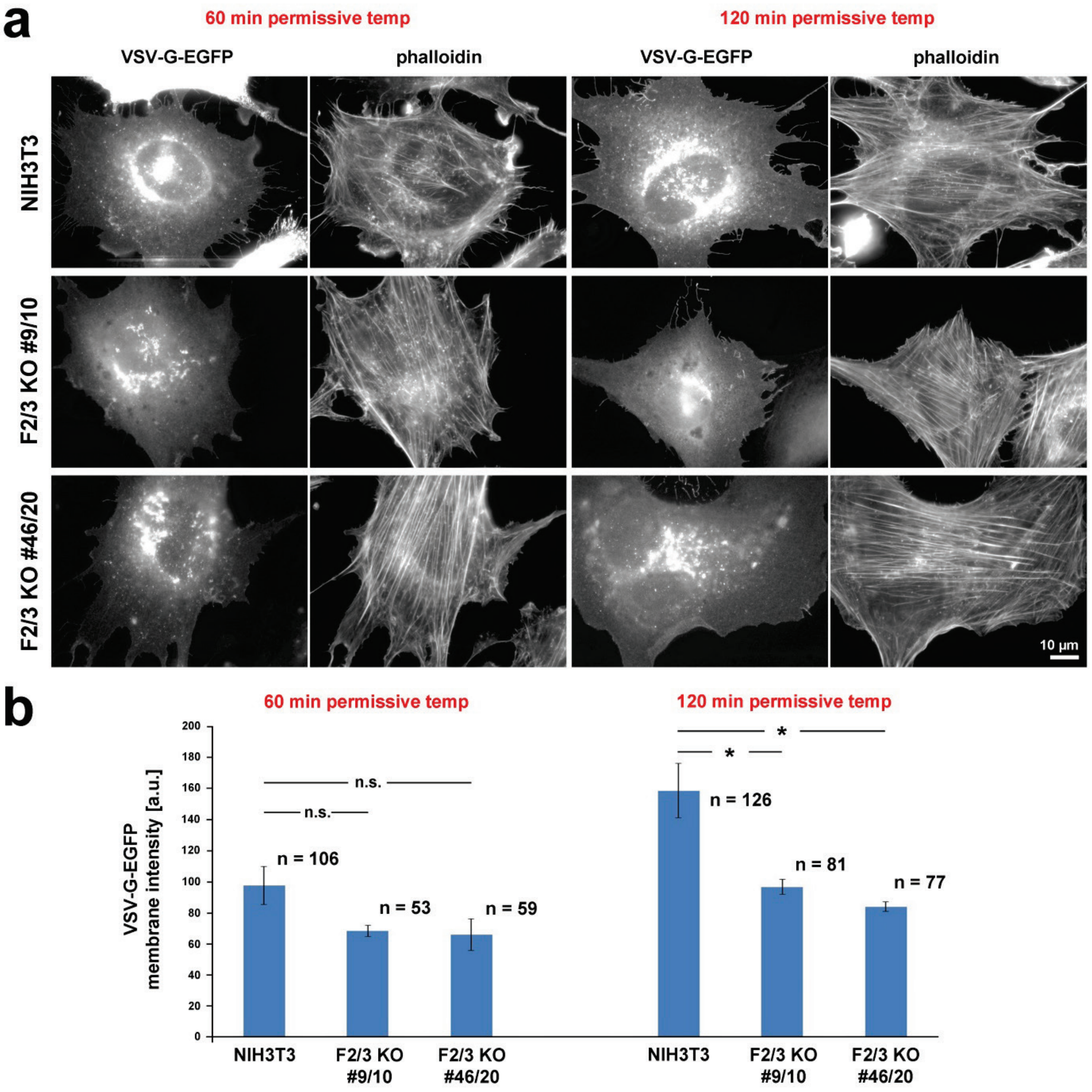

Figure S21: Plasma membrane levels of VSV-G-EGFP are reduced in FMNL2/3 KO cells.

(**a**) Representative examples of control and FMNL2/3 KO cells illustrating VSV-G-EGFP signals in the plasma membranes of respective cells switched to permissive temperature for indicated times. (**b**) Quantitative analysis of average cell surface surface intensities of VSV-G-EGFP (arbitrary units) at 60 and 120 min after shift to 32°C (permissive temperature) for control and FMNL2/3 KO cells, as indicated. Data are arithmetic means and ± sem from four independent experiments, n equals cell numbers analyzed; statistics was done using non-parametric Mann-Whitney rank sum test. Note plasma membrane association of VSV-G-EGFP to be reduced in both FMNL2/3 double KO clones in a statistically significant fashion 2h after temperature shift.

SUPPLEMENTARY MOVIE LEGENDS

Supplementary Movie 1

FMNL2-EGFP co-localizes with Cdc42 in the perinuclear region. 3D-projection of a reconstructed stack of 3D-Structured Illumination Microscopy (3D-SIM) images of a B16-F1 cell transfected with FMNL2-EGFP (green) and mCherry-Cdc42-L61 (red). Note the prominent overlap of these proteins in the perinuclear region, likely representing the Golgi.

Supplementary Movie 2

FMNL2-EGFP accumulates at Golgi structures. Animation of a volume view of a reconstructed stack of 3D-Structured Illumination Microscopy (3D-SIM) images of a COS-7 cell transfected with FMNL2-EGFP (green) and counterstained for GM130 (red).

Supplementary Movie 3

FMNL3-EGFP accumulates at Golgi structures. Animation of a volume view of a reconstructed stack of 3D-Structured Illumination Microscopy (3D-SIM) images of a COS-7 cell transfected with FMNL3-EGFP (green) and counterstained for GM130 (red).

Supplementary Movie 4

FMNL2-EGFP associates with intracellular vesicles. Time-lapse fluorescence and phase contrast microscopy of B16-F1 cell migrating on laminin and transiently transfected with FMNL2-EGFP, accumulating at lamellipodia and filopodia tips, as expected. Additionally, however, FMNL2-EGFP targeted to various, highly dynamic vesicles displaying typical, saltatory and bouncing movements within the cell. Time is given in minutes and seconds. Scale bar corresponds to 10 µm.

Supplementary Movie 5

FMNL3-EGFP accumulates at intracellular vesicular structures. A B16-F1 cell migrating on laminin and transiently expressing FMNL3-EGFP was recorded by time-lapse fluorescence microscopy and phase contrast optics, as indicated. In analogy to FMNL2-EGFP, FMNL3-EGFP displays prominent accumulation at highly dynamic vesicles. Time is given in minutes and seconds. Scale bar, 10 µm.

Supplementary Movie 6

Morphology of the *cis*-Golgi in NIH control fibroblast, example 1.

Animated 3D-SIM volume view of a reconstructed stack of an NIH3T3 cell stained for the *cis*-Golgi marker GM130. Note that visible Golgi cisternae are mostly connected, building continuous Golgi ribbons.

Supplementary Movie 7

Morphology of the *cis*-Golgi in NIH control fibroblast, example 2. For legend see Supplementary Movie 6.

Supplementary Movie 8

FMNL2/3 removal causes a fragmented Golgi morphology, clone #9/10, example 1. Representative, animated 3D-SIM volume view of a reconstructed stack of a cell representing NIH3T3 clone #9/10, genetically deleted for FMNL2 and -3 and stained for the *cis*-Golgi marker GM130. Note that the *cis*-Golgi compartment appears highly fragmented as compared to control NIH 3T3 cells (compare Supplementary Movies 6 and 7).

Supplementary Movie 9

FMNL2/3 removal causes a fragmented Golgi morphology, clone #9/10, example 2. For legend see Supplementary Movie 8.

Supplementary Movie 10

FMNL2/3 removal causes a fragmented Golgi morphology, clone #46/20, example 1. Representative, animated 3D-SIM volume view of a reconstructed stack of a cell representing NIH3T3 clone #46/20, genetically deleted for FMNL2 and -3 and stained for the *cis*-Golgi marker GM130. Note that the *cis*-Golgi compartment appears highly fragmented as compared to control NIH 3T3 cells (compare Supplementary Movies 6 and 7).

Supplementary Movie 11

FMNL2/3 removal causes a fragmented Golgi morphology, clone #46/20, example 2. For legend see Supplementary Movie 10.

Supplementary Movie 12

FMNL2-EGFP dynamics during lamellipodium protrusion and membrane ruffling. Time-lapse epifluorescence imaging of NIH 3T3 cell plated on fibronectin and transiently co-expressing FMNL2-EGFP and constitutively active, mCherry-tagged Cdc42-L61. The panel on the right shows FMNL2-

EGFP signal in higher magnification (inset) in the region depicted by the white rectangle on the left. Note that at variance to mCherry-Cdc42, FMNL2-EGFP is associated with the plasma membrane during ruffling protrusion, and frequently arises as cytoplasmic spots upon folding backwards of the ruffing membrane, suggesting the formin to be taken up and recycled or degraded by this process. Time is given in minutes and seconds. Scale bar, 10 µm, and in inset, 3 µm.
